# Efficient Gene Knock-out and Knock-in with Transgenic Cas9 in *Drosophila*

**DOI:** 10.1534/g3.114.010496

**Published:** 2014-03-21

**Authors:** Zhaoyu Xue, Mengda Ren, Menghua Wu, Junbiao Dai, Yikang S. Rong, Guanjun Gao

**Affiliations:** * School of Life Sciences, Tsinghua University, Beijing 100084, China; † Laboratory of Biochemistry and Molecular Biology, National Cancer Institute, National Institutes of Health, Bethesda, Maryland 20892

**Keywords:** knock-out, knock-in, Cas9, gRNA, *Drosophila*

## Abstract

Bacterial Cas9 nuclease induces site-specific DNA breaks using small gRNA as guides. Cas9 has been successfully introduced into *Drosophila* for genome editing. Here, we improve the versatility of this method by developing a transgenic system that expresses Cas9 in the *Drosophila* germline. Using this system, we induced inheritable knock-out mutations by injecting only the gRNA into embryos, achieved highly efficient mutagenesis by expressing gRNA from the promoter of a novel non-coding RNA gene, and recovered homologous recombination-based knock-in of a fluorescent marker at a rate of 4.5% by co-injecting gRNA with a circular DNA donor.

The RNA-guided CRISPR/Cas9 system has been successfully used for the purpose of genome editing in many organisms, including *Drosophila* (Golic 2013). Cas9 nuclease is guided to its target DNA by a small guide RNA (gRNA), which shares a homologous region of approximately 20 nucleotides (nt) with the cleavage site. By direct injection of synthesized Cas9 mRNA and customized gRNA into wild-type *Drosophila* embryos, we previously achieved a mutational rate of up to 100% in the germline (Yu *et al.* 2013). We considered that at least two aspects of our previous technique could be improved upon. First, the generation and handling of *in vitro* synthesized RNA, particularly the relatively large Cas9 mRNA, could be challenging to inexperienced researchers, which could result in inconsistencies in mutational efficiency. Second, the utility of Cas9 in generating knock-in mutations has not been adequately demonstrated in *Drosophila*. Here, we report efficient knock-out and knock-in manipulations of the *Drosophila* genome with a Cas9 transgenic system. We note that several groups have independently developed tools to conduct mutagenesis using transgenic lines expressing Cas9 in the germline (Sebo *et al.* 2014; Ren *et al.* 2013; Kondo and Ueda 2013) and to generate knock-in mutations (Baena-Lopes *et al.* 2013; Gratz *et al.* 2014). In addition, an efficient knock-in system using TALEN has been developed (Katsuyama *et al.* 2013).

## Materials and Methods

### Fly stocks

Several different wild-type stocks such as *w^1118^*, *y w* and Oregon-R (red eye) were used in this study. All balancers were obtained from the Bloomington Stock Center (Indiana). Flies were cultured at 25°.

### Plasmid construction (PiggyBac-*vasa*-cas9, pUAST-gRNA, and homologous recombination donor plasmid)

#### PiggyBac-vasa-cas9 construction:

PiggyBac-*vasa*-cas9 construction required three steps. First, a standard entry vector with a visible GFP marker, PiggyBac-9A-GFP, was constructed by inserting a 3×P3GFP fragment with SV40 3′-UTR from the plasmid p3×P3GFP (stock in our laboratory) between the *Xma*I and PspomI sites of PiggyBac-9A(Venken *et al.* 2006). Second, a 2241-bp promoter sequence and a 576-bp 3′-UTR-containing sequence were amplified from the vasa gene by PCR and were cloned into the same *pEASY-T* vector (Transgen, Beijing) to generate the germline-expression vector TA-*vasa*Pro-UTR. The cas9-gene fragment was released from pSP6-2NLS-spcas9 (stock in our laboratory) by *Not*I/*Sal*I and inserted between the *Not*I and *Xho*I sites of TA-*vasa*Pro-UTR to generate TA-*vasa*Pro-cas9-UTR. Eventually, the larger fragment containing *vasa*Pro-cas9-UTR was released from TA-*vasa*Pro-cas9-UTR by *Xma*I/*Spe*I and cloned into PiggyBac-9A-GFP using the same restriction enzymes. This produced the final transgenic vector PiggyBac-*vasa*-GFP (Figure S6).

#### pUAST-gRNA construction:

Three steps were needed for pUAST-gRNA construction. First, a fragment containing 3×P3RFP and the *tubulin* 3′ UTR was amplified from the pM3×P3-RFPattP′ vector (Bischof *et al.* 2007) and used to replace the *white* gene between two *Eco*RV sites in pUAST; this created the entry plasmid pUAST-RFP. Second, a 400-bp *Drosophila* U6B promoter sequence (Wakiyama *et al.* 2005) and the 1133-bp promoter sequence from *Drosophila* non-coding RNA CR34335 (Graveley *et al.* 2010) (Figure S4) were separately cloned into the pMD19-TA gRNA scaffold vector (stock in our laboratory) to create TA-U6B-gRNA and TA-CR7T-gRNA, respectively. The KOD mutagenesis kit (TOYOBO, Japan) was used to insert 20-bp target sequences between the promoter and the gRNA scaffold. Third, the target-specific U6B/CR7T-gRNAs were released by *Spe*I/*Kpn*I and cloned using the same sites in pUAST-RFP, generating the final transgenic gene-specific vectors (Figure S6). In total, five transgenic gRNA vectors were generated: pUAST-U6B-k81-gRNA for targeting the *ms(3)k81* locus; pUAST-U6B-y1-gRNA and pUAST-CR7T-y2-gRNA for targeting the *yellow* locus; and pUAST-CR7T-w1-gRNA and pUAST-CR7T-w2-gRNA for targeting the *white* locus. All primers are listed in Table S2 and Table S3.

#### Donor plasmid pHisc-RA-RFP construction:

To generate the pHisc-RA-RFP donor plasmid, a synthetic DNA fragment containing the attP, FRT, and 3×P3RFP sequences was cloned into *pEASY-T* to generate vector *pEASY*-RFP (available upon request). Subsequently, 1.5-kb and 1.2-kb sequences, respectively, corresponding to the 5′ and 3′ ends of the histone cluster right-arm (Hisc-RA) were inserted flanking the RFP cassette to obtain the final construct pHisc-RA-RFP.

### gRNA injections

DNA templates were PCR-amplified from gRNA scaffold vectors for *in vitro* gRNA transcription (Yu *et al.* 2013). Transcription was performed with the RiboMAX Large Scale RNA Production Systems-T7 Kit (Promega, USA) according to the manufacturer’s protocol. Purified gRNA (0.2 μg/μl) was injected into *vasa*-Cas9 fly embryos either directly or after mixing with purified donor plasmid (0.8 μg/μl) according to standard procedures. All primers are listed in Table S1.

### Fly transformation and genetics

To obtain transgenic *vasa*-Cas9 flies, the PiggyBac-*vasa*-cas9 vector was mixed with helper vector PiggyBac-transposase (Thibault *et al.* 2004) and injected into *w^1118^* fly embryos. Flies carrying *vasa*-Cas9 were identified by GFP expression in the eye when viewed under a fluorescence stereomicroscope. Mutation position was mapped to chromosome 2 after crossing with balancer flies, and homozygous *vasa*-Cas9 flies were established by subsequent crossing.

To obtain transgenic gRNA flies, the five pUAST-U6B/CR7T-gRNA vectors were separately injected into *w^1118^* fly embryos, and at least three independent lines per transgenic gRNA were identified through observation of the RFP eye marker. The vectors targeted the following loci: pUAST-U6B-k81-gRNA, the *ms(3)k81* locus; pUAST-U6B-y1-gRNA and pUAST-CR7T-y2-gRNA, the *yellow* locus; and pUAST-CR7T-w1-gRNA and pUAST-CR7T-w2-gRNA, the *white* locus. After transgenic gRNA flies were crossed with the *vasa*-Cas9 flies, F_0_ flies expressing both fluorescent markers were individually collected and were crossed to wild-type flies to generate F_1_ germline transformants. F_0_ flies and F_1_ progeny were phenotypically and genotypically assessed. For the *vasa*-Cas9 fly embryos directly injected with gRNA, F_0_ and F_1_ were tested; however, for embryos receiving coinjections of gRNA and donor plasmid, only F_1_ flies with visible RFP eye markers were used for PCR analysis and DNA sequencing.

### Screening and analysis of mutations formed via nonhomologous end joining

F_0_ (from injected embryos) and F_1_ (progeny of F_0_) *ms(3)k81*-disrupted flies that resulted from direct injection of synthetic gRNA into *vasa*-Cas9 fly embryos were recovered and analyzed as described previously (Yu *et al.* 2013). All five transgenic pUAST-gRNA lines were crossed with *vasa*-Cas9 flies to induce F_0_ mutations at the three loci of interest (*ms(3)k81*, *white*, and *yellow*). Each F_0_ fly containing transgenic *vasa*-Cas9 and gRNA was then crossed to wild-type flies to recover F_1_ progeny with mutations resulting from germline transmission. For the *ms(3)k81* locus, mutations of each F_0_ fly containing transgenic *vasa*-Cas9/ pUAST-U6B-k81-gRNA and its F_1_ offspring were recovered and analyzed as described above. In case of the *yellow* locus, pUAST-U6B-y1-gRNA and pUAST-CR7T-y2-gRNA were used. The target *yellow* genomic region was amplified by PCR from a single transgenic *vasa*-Cas9/pUAST-gRNA F_0_ fly using appropriate primers (Table S3). The corresponding PCR products were digested with T7 endonuclease I (New England Biolabs, USA) and analyzed by gel electrophoresis (Kondo and Ueda 2013). The mosaic yellow phenotype evaluation in F_0_ transgenic *vasa*-Cas9/pUAST-gRNA and indel scoring in F_1_ progeny were performed according to previous methods (Liu *et al.* 2012). For the *white* locus, two pUAST-gRNA lines (pUAST-CR7T-w1-gRNA and pUAST-CR7T-w2-gRNA) were used to induce the germline transmission mutations. First, a *vasa*-Cas9 fly expressing the *white* gene on the X-chromosome was identified from the progeny of Oregon-R flies and *vasa*-Cas9 flies by screening for red eyes with GFP expression. The female red-eye *vasa*-Cas9 flies were crossed to pUAST-CR7T-gRNA males to generate male F_0_ flies, which were identified by screening for the GFP and RFP eye markers. The male F_0_ flies were then crossed to wild-type flies to obtain F_1_ progeny with germline-transmitted mutations. The mosaic eye phenotype of F_0_ transgenic *vasa*-Cas9/pUAST-gRNA was investigated and indel scoring in F_1_ progeny was performed and analyzed as above. Primers used for PCR and sequencing are listed in Table S3.

### Recovery and analysis of germline mutants via homologous recombination

**Figure 1:**
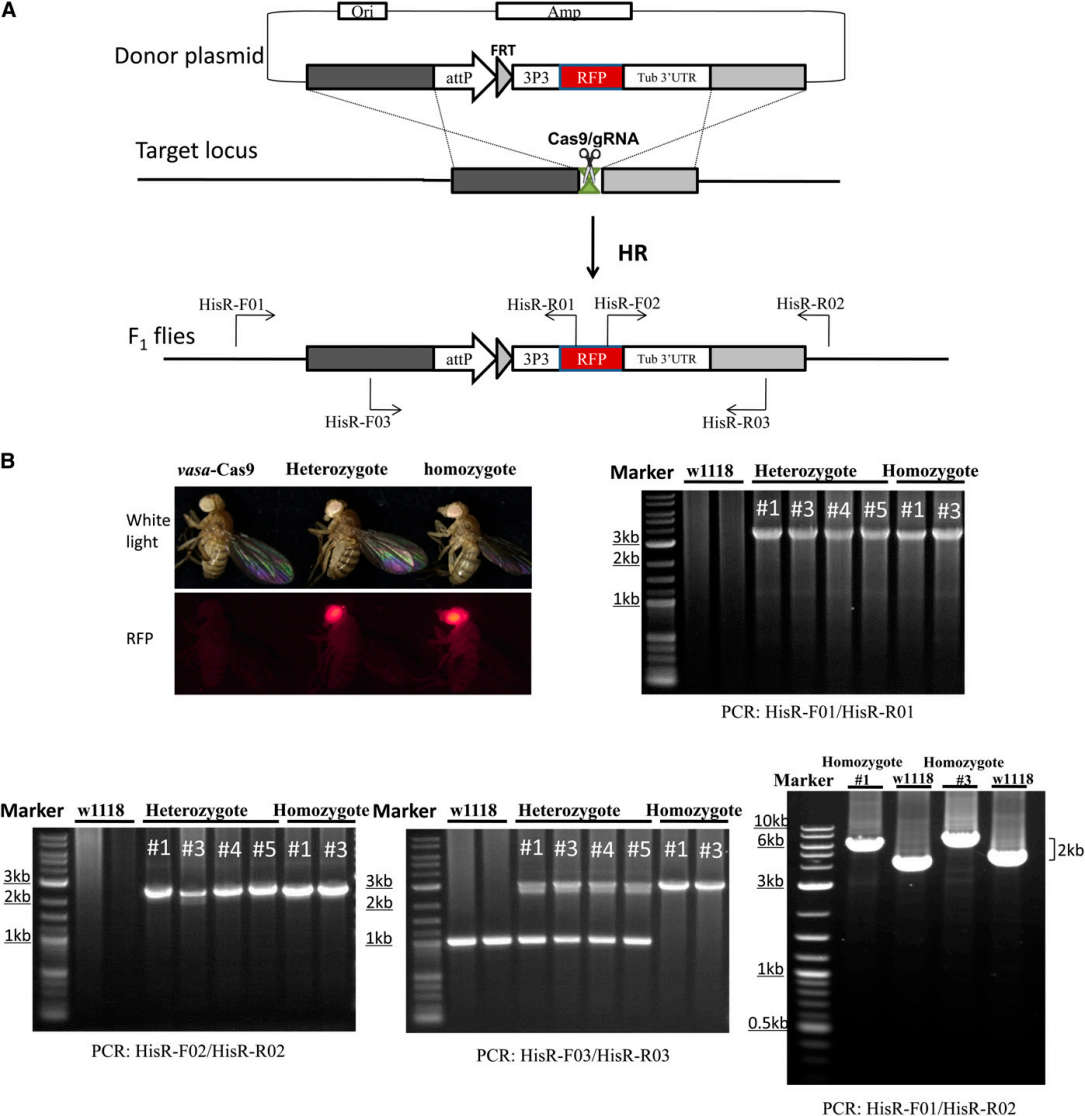
Knock-in of a marker gene using transgenic Cas9/gRNA system. (A) Strategy for knocking-in an attP-FRT-RFP cassette to the right of the *histone* locus. The double-strand break induced at the target locus is repaired by HR between the exogenous donor (top) and the target locus (middle), leading to precise insertion of the heterogeneous cassette (bottom). Three different primer pairs were designed for molecular analysis (B). The image at the top left shows examples of flies expressing the RFP eye-marker. The other four images illustrate the results of PCR genotyping in heterozygous and homozygous RFP flies. There is a 2-kb difference in size of PCR products from the wild-type control *vs.* RFP-expressing flies, indicating the successful insertion of the attP-FRT-RFP cassette.

Hisc-RA-gRNA and the donor pHisc-RA-RFP plasmid were coinjected into *vasa*-Cas9 fly embryos, and germline mutations containing the attP-FRT-RFP fragment were recovered by collecting F_1_ progeny with visible RFP eye expression. Genomic DNA was prepared from individual heterozygous and some homozygous candidates. The target genome region was amplified using three pairs of primers, including one pair that flanked the locus (His-F01 and His-R02) (Figure 1A). The corresponding products were analyzed by gel electrophoresis and sequenced (Figure 1B). Homozygous knock-in stocks were established after crossing to balancer flies. Primers used for PCR and sequencing are listed in Table S3.

## Results and Discussion

### Cas9 knock-out by gRNA injection into Cas9-producing embryos

To eliminate the need to synthesize Cas9 mRNA for injection, we set out to achieve Cas9 expression in the germline by driving the cas9 gene with the *vasa* promoter (Lasko and Ashburner 1988). We recovered a single piggyBac-mediated insertion of this transgene in chromosome 2. Transgenic Cas9 flies are homozygous viable and fertile. To investigate whether these transgenic flies can be used to efficiently produce site-specific mutations, we set out to knock-out *ms(3)k81* using a previously designed gRNA (Yu *et al.* 2013). Mutations in *ms(3)k81* cause male sterility (Fuyama 1986). When we injected Cas9 mRNA and *ms(3)k81* gRNA simultaneously, all surviving males were sterile and all harbored somatic *ms(3)k81* mutations (Yu *et al.* 2013). We hypothesized that in these males, both copies of the *ms(3)k81* gene might have been mutated in male germ cells. Many surviving females were fertile, and all of their progeny were mutants for *ms(3)k81*, consistent with our proposition that both copies of *ms(3)k81* had been mutated as a result of the repair of a Cas9-induced DNA break.

Here, we injected only the *ms(3)k81* gRNA into vasa-cas9 embryos. We detected somatic *ms(3)k81* mutations in 70% of the surviving males (12/17). All 12 males were fertile and produced a *ms(3)k81* mutational rate of 25% in the germline (Table 1). Surviving females produced a similar mutational rate in their germline (Table 1). Therefore, our transgenic system can efficiently induce mutations in the germline (Figure S1A).

**Table 1:** Frequencies of *vasa*-Cas9/gRNA–mediated mutagenesis

**Locus**	**gRNA**	**F_0_**	**Fertile F_0_ with Somatic Mutation *n/n*, (%)**	**F_1_ Mutation Rates *n/n*, (%)**
*ms(3)k81*	Injection	Female	20/26 (76.9)	11/49 (22.4)
		Male	12/17 (70.1)	5/20 (25.0)
	U6B-k81	Female	22/22 (100)	4/28 (14.3)
		Male	26/26 (100)*^a^*	NA
*Yellow*	U6B-y1	Female	36/36 (100)	221/588 (37.6)
		Male	8/8 (100)	158/178 (88.8)*^b^*
	CR7T-y2	Female	30/30 (100)	593/628 (94.4)
		Male	7/7 (100)	74/82 (90.2)*^b^*
*White*	CR7T-w1	Female	23/23 (100)	NA
		Male	17/17 (100)	430/664 (64.8)
	CR7T-w2	Female	23/23 (100)	NA
		Male	18/18 (100)	86/506 (17.0)

NA, not applicable.

a All males are sterile.

b The female progeny of F_1_ was counted as total progeny.

### Knock-out by transgenic expression of both gRNA and Cas9

To completely eliminate the need to inject RNA, we set out to establish a transgenic gRNA system to combine with germline Cas9 expression. The U6B promoter has commonly been used for gRNA expression (Kondo and Ueda 2013; Ren *et al.* 2013; Sebo *et al.* 2014). Here, we were interested in testing the efficacy of using the promoter of another non-coding RNA gene to drive gRNA expression. We chose the non-coding *CR34335* gene, which is ubiquitously expressed at high levels based on genome-wide RNA-seq data (Graveley *et al.* 2010), and established a gRNA expression system that is driven by the CR34335 promoter, which we named CR7T. gRNA for various targets were put under the control of the U6B or CR7T promoters and introduced into the fly genome by *P*-element–mediated transformation.

As a test of the functionality of our U6B expression system, we generated flies with vasa-Cas9 and U6B-k81, which expresses a gRNA designed for the *ms(3)k81* used previously. All males (n = 26) were sterile, suggesting that both *ms(3)k81* copies had been mutated in male germ cells. Twenty-two females were fertile, from which we obtained a *ms(3)k81* mutational rate of 14.3% (Table 1 and Figure S1B). Therefore, we observed a lower efficiency using the U6B promoter in the female *vs.* the male germline. We tested the same system on the *yellow* locus and observed a similar effect (y1 gRNA in Table 1). In contrast, for the y2 gRNA against *yellow*, we achieved equally high (more than 90%) frequencies from both germlines using the CR7T promoter (Table 1 and Figure S2). We further confirmed the utility of CR7T in driving gRNA expression by using it in the generation of mutations at two target locations in *white*, both with reasonably high efficiencies (Table 1 and Figure S3).

In summary, we successfully generated knock-out mutations to three genes by transgenically expressing both Cas9 and gRNA. The germline mutational rate ranged from 14% to 95%. Additionally, we developed a new gRNA expression system using the CR7T promoter.

### Knock-in by Cas9-induced homologous recombination

The ability to introduce predefined genetic modifications is highly desirable in genome editing. The knock-in approach utilizes the ability of the cell to incorporate exogenously supplied DNA into the host genome by homologous recombination (HR). Previously, a 50-nt modification (an attP attachment site for the phiC31 integrase) was successfully introduced into the *yellow* locus by Cas9-induced HR (Gratz *et al.* 2013). This modification was accomplished by co-injection of Cas9 mRNA, gRNAs against *yellow*, and single-stranded oligos as donor DNA for recombination [single-strand oligodeoxynucleotide (ssODN)]. The ssODN had two 60 nt homology arms flanking attP.

For the Cas9-induced knock-in method to become routine practice in *Drosophila*, at least two issues need to be further examined. The first issue is whether this approach can be applied to a general locus where an easily screenable phenotype (such as yellow body color in *yellow*-mutant flies) is not available. The second issue is whether double-stranded donor DNA can be used when the size of the desired modification precludes the use of oligos. As an attempt to address the first question, we used ssODN to introduce an *FRT* site along with an *attP* to the left side (centromere-distal) of the histone locus on chromosome 2. The oligo is 135 nt in total length with a 24-nt homology arm on the left side and a 27-nt homology arm on the right side. Using PCR as a screening method, we successfully recovered precise knock-in events in 10 of the 112 F_1_ progeny screened, suggesting that the ssODN approach can, in principle, be applied without the necessity of a screenable phenotype in the target locus. PCR followed by sequencing confirmed the precise integration of the *FRT* and *attP* elements (Figure S5). However, a similar approach applied to the right (centromere-proximal) side of the histone locus failed, with the oligo being 87 nt in total length, with a 26-nt homology arm on left side and a 27-nt homology arm on the right side. The reason for this is unclear.

As an attempt to address the second question, we modified the histone cluster locus using a plasmid as the HR donor. The overall scheme of this targeting experiment is shown in Figure 1A. Our goal was to knock-in an element with attP and FRT along with a 3×P3RFP marker gene to the right side of the histone locus. In the donor plasmid, these three DNA elements were flanked by a 1.5-kb homology arm to the left and a 1.2-kb arm to the right (Figure 1A). The inclusion of these elements into the donor also disrupted a Cas9 cleavage site so that Cas9 will only cut at the chromosome but not the donor. The *in vitro* synthesized gRNA molecules and donor DNA were injected into vasa-Cas9 embryos. We set-up 89 fertile crosses from flies that survived the injection process. Using the RFP marker, we recovered at least four independent events with a total of 49 F_1_ progeny showing RFP expression. PCR amplification followed by sequencing confirmed that in all of these 49 F_1_ individuals, a single copy of the 2-kb RFP cassette was precisely targeted to the desired location (Figure 1B). Therefore, we have demonstrated the targeted insertion of a large piece of heterologous DNA (2 kb in total) aided by the use of a visible marker in *Drosophila*. Using this RFP-marked insertion and the previously generated attP-FRT insertion to the left of the histone locus, we succeeded in deleting the entire 125-kb histone locus using FLP-mediated recombination (data not shown).

## 

## Supplementary Material

Supporting Information
