# Supplementary material for: Efficient Gene Knock-out and Knock-in with Transgenic Cas9 in Drosophila
Source: G3 (Bethesda). 2014 Mar 21;4(5):925–9. doi: 10.1534/g3.114.010496 (PMC4025491; doi:10.1534/g3.114.010496)
Supplement: Supporting Information [file supp_4_5_925__index.html]

Efficient Gene Knock-out and Knock-in with Transgenic Cas9 in Drosophila — Supporting Information 

# Efficient Gene Knock-out and Knock-in with Transgenic Cas9 in *Drosophila*

## Supporting Information for Xue *et al.*, 2014

**Files in this Data Supplement:**

- Supporting Information - Figures S1-S6 and Tables S1-S3 (PDF, 1 MB)
- Figure S1 - (A) Mutations induced by injection of k81-gRNA in transgenic vasa-Cas9 embryos. (B) Mutations induced by transgenic vasa-Cas9/k81-gRNA at *ms(3)k81*. (PDF, 365 KB)
- Figure S2 - (A) T7 endonuclease I (T7E1) assay of mutation at *yellow* locus induced by transgenic vasa-Cas9/yw-gRNA (F0 flies). (B) Indel mutations induced by transgenic vasa-Cas9/pyw-gRNA at *yellow* locus. (PDF, 410 KB)
- Figure S3 - Indel mutations induced by transgenic vasa-Cas9/w-gRNA at *white* locus. (PDF, 212 KB)
- Figure S4 - The DNA sequences of U6B promoter and CR34335 promoter used in our study. (PDF, 238 KB)
- Figure S5 - The DNA sequence of the attP-FRT-RFP cassette in our study. (PDF, 310 KB)
- Figure S6 - Maps of the plasmids with vasa-cas9 or the U6B/CR7T promoters. (PDF, 176 KB)
- Table S1 - *Drosophila* gene sites targeted in this study. (PDF, 127 KB)
- Table S2 - (I) List of primers for vasa-Cas9/pUAST-gRNA vector constructions used in this study. (II) List of primers for transgenic gRNA vectors constructions used in this study. (PDF, 118 KB)
- Table S3 - List of primers for PCR check in mutations used in this study. (PDF, 116 KB)
