# Supplementary material for: Efficient Gene Knock-out and Knock-in with Transgenic Cas9 in Drosophila
Source: G3 (Bethesda). 2014 Mar 21;4(5):925–9. doi: 10.1534/g3.114.010496 (PMC4025491; doi:10.1534/g3.114.010496)
Supplement: Supporting Information [file supp_g3.114.010496_010496SI.pdf]

G3· Genes | Genomes | Genetics

Efficient gene knock-out and knock-in with transgenic Cas9 in *Drosophila*

Zhaoyu Xue[1], Mengda Ren[1], Menghua Wu[1], Junbiao Dai[1], Yikang S. Rong[2], and Guanjun Gao[1,*]

[1] School of Life Sciences, Tsinghua University, Beijing 100084, China
[2]Laboratory of Biochemistry and Molecular Biology, National Cancer Institute, National Institutes of Health, Bethesda, MD 20892, USA

[*]Corresponding author:
Guanjun Gao, School of Life Sciences, Tsinghua University, Yuanmingyuan Road 1, Beijing 100084, China. E-mail: gaogu@mail.tsinghua.edu.cn

DOI: 10.1534/g3.114.010496

A

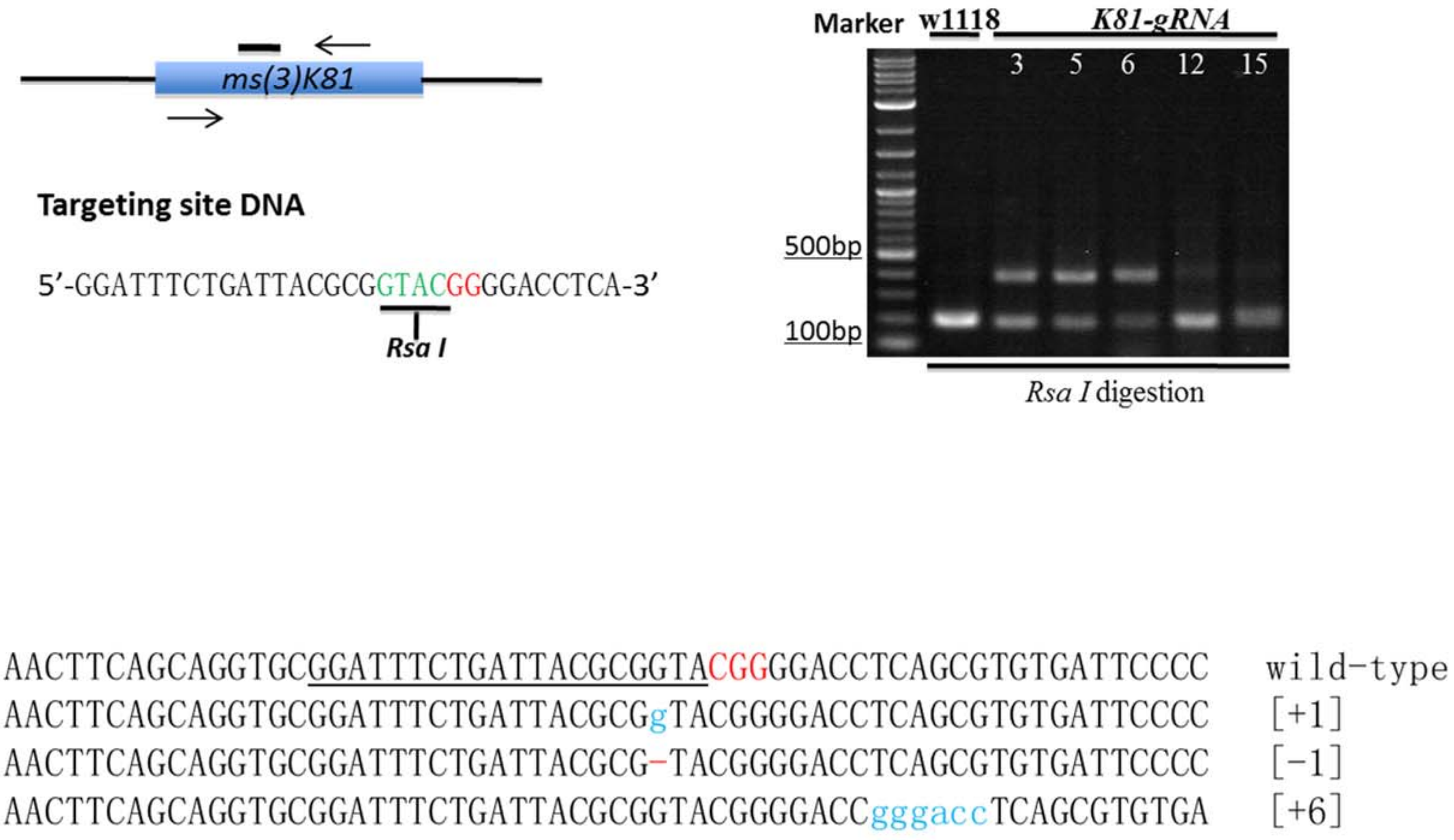
ms(3)K81
Targeting site DNA
5'-GGATTTCTGATTACGCGGTACGGGGACCTCA-3'
Rsa I
Marker
w1118
K81-gRNA
3
5
6
12
15
500bp
100bp
Rsa I digestion
AACTTCAGCAGGTGCGGATTTCTGATTACGCGGTACGGGGACCTCAGCGTGTGATTCCCC wild-type
AACTTCAGCAGGTGCGGATTTCTGATTACGCGgTACGGGGACCTCAGCGTGTGATTCCCC [+1]
AACTTCAGCAGGTGCGGATTTCTGATTACGCG-TACGGGGACCTCAGCGTGTGATTCCCC [-1]
AACTTCAGCAGGTGCGGATTTCTGATTACGCGGTACGGGGACCgggaccTCAGCGTGTGA [+6]

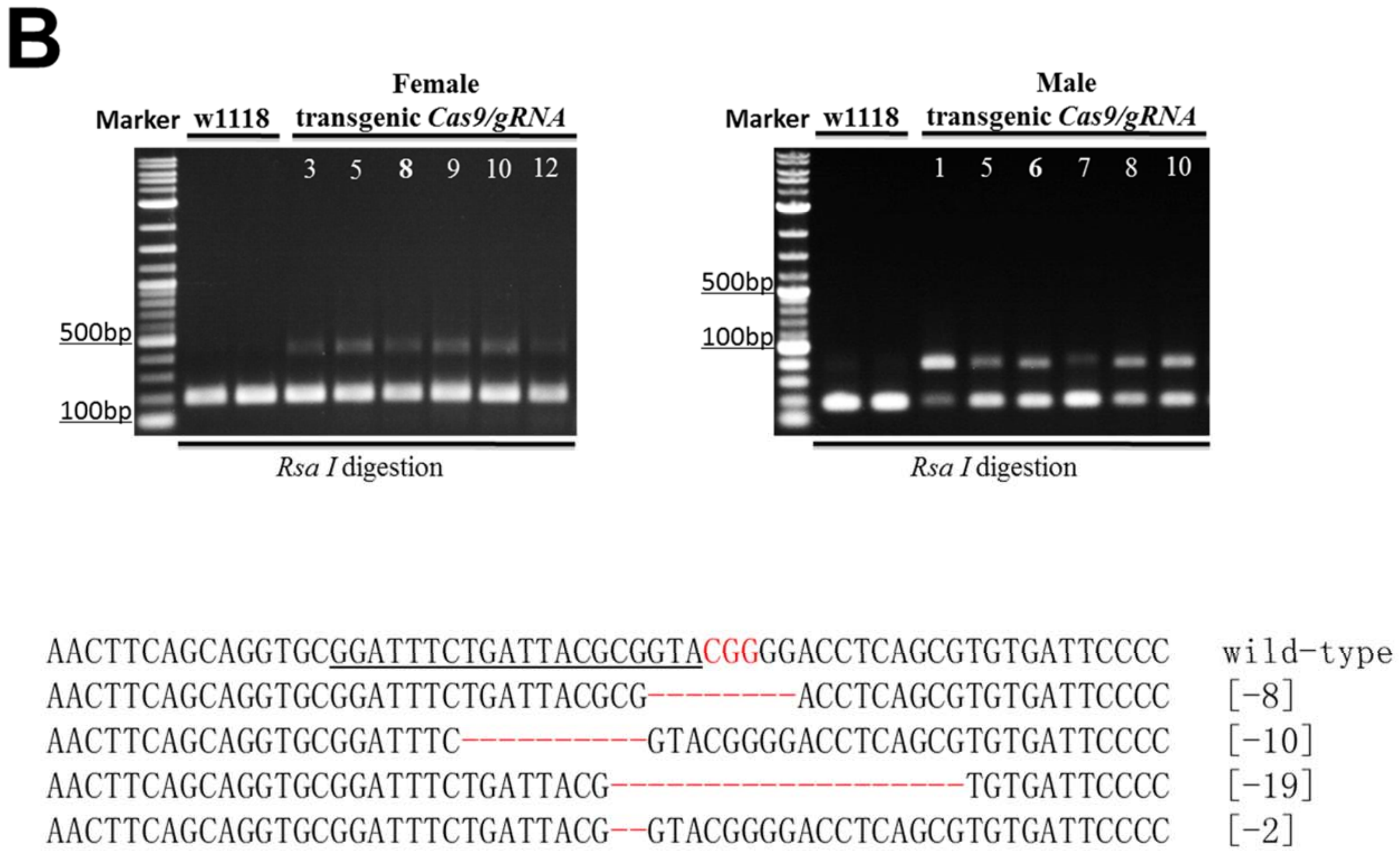

Figure S1 (A) Mutations induced by injection of k81-gRNA in transgenic vasa-Cas9 embryos. Images on the top left corner of each panel show targeting site. Enzyme cutting sites chosen for identification of mutations are underlined. Images on each top right corner show the enzyme digestion results of PCR products of wild-type and transgenic Cas9/gRNA-induced F_0 mutants. Representative DNA sequencing results of the PCR products from F_1 individual flies show indel mutations induced by transgenic Cas9/gRNA at the targeted *ms(3)k81* locus. The wild-type DNA sequence is shown on the top with the target site underlined and the PAM sequence highlighted in red. Deletions are shown as red dashes and insertions highlighted in blue and lowercase letters. The change of DNA length (in nucleotides) in each mutation is indicated to the right of each sequence (+, insertion; -, deletion). (B) Mutations induced by transgenic vasa-Cas9/k81-gRNA at *ms(3)k81*. Upper pictures show the enzyme digestion results of PCR products of wild-type and transgenic Cas9/gRNA-induced F_0 mutants. Lower pictures show representative DNA sequencing results of the PCR products from F_1 individual flies showing indel mutations induced by transgenic Cas9/gRNA at the targeted *ms(3)k81* locus.

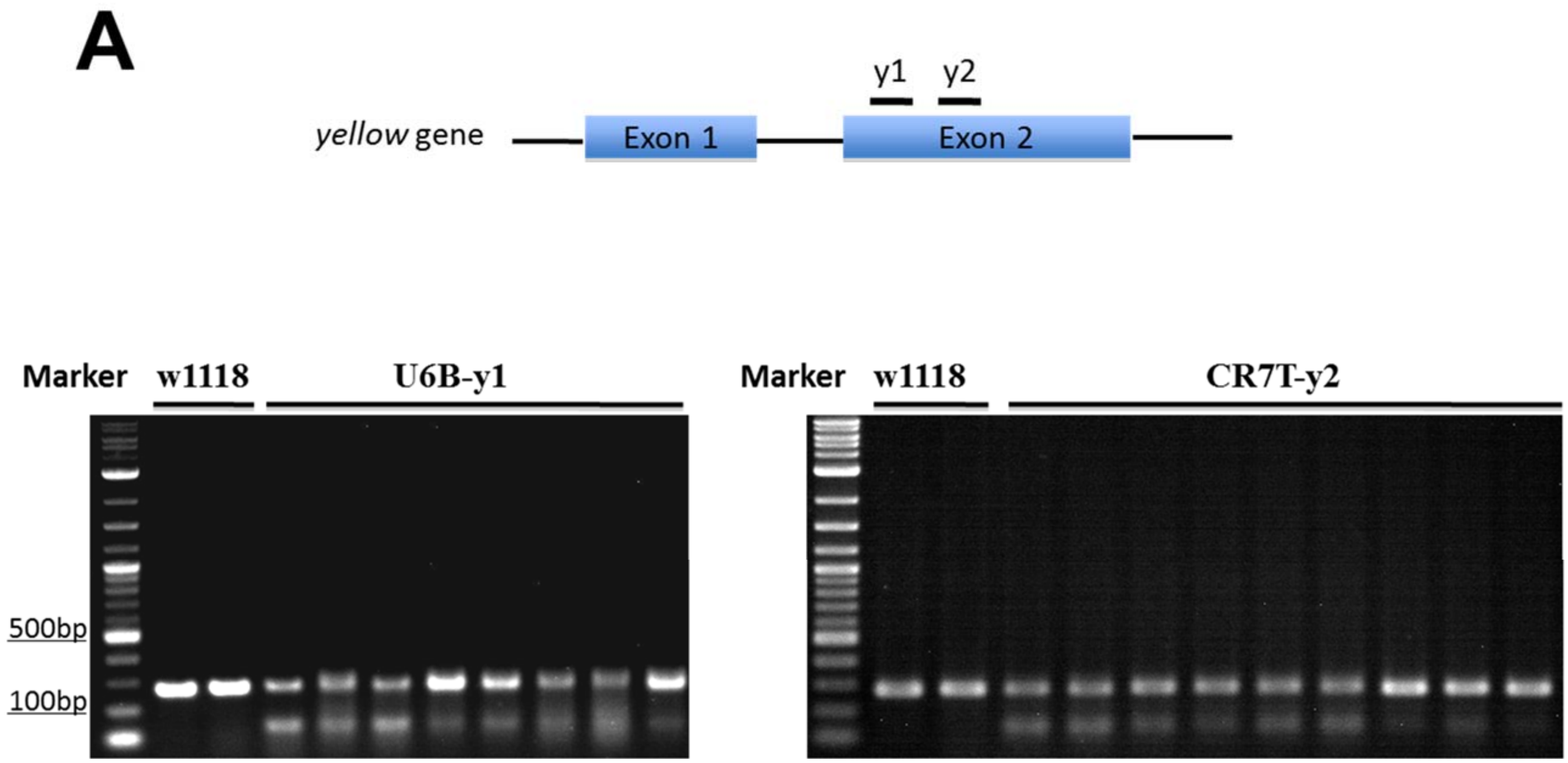
A
y1
y2
yellow gene
Exon 1
Exon 2
Marker
w1118
U6B-y1
500bp
100bp
Marker
w1118
CR7T-y2

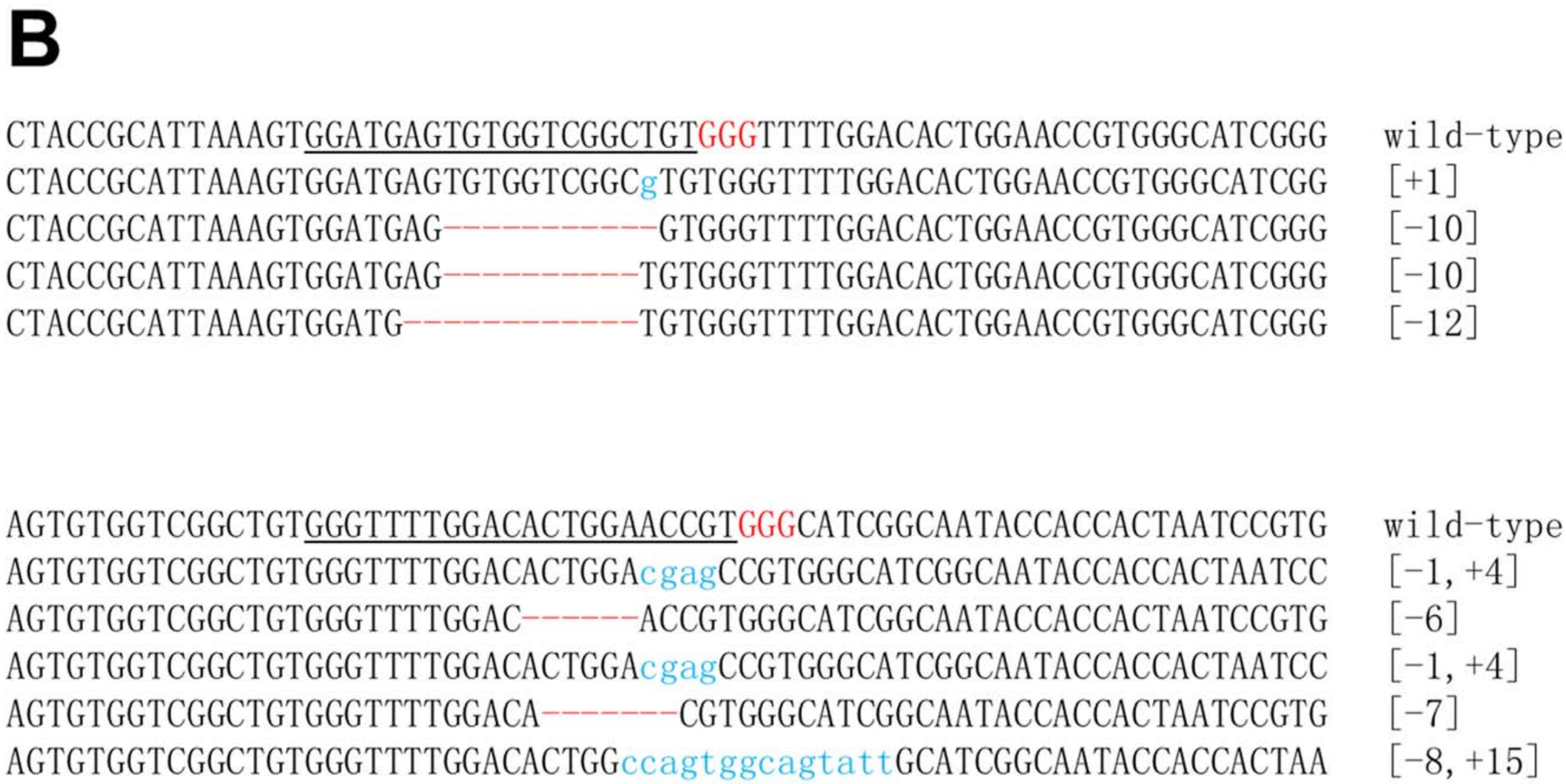

Figure S2 (A) T7 endonuclease I (T7E1) assay of mutation at *yellow* locus induced by transgenic vasa-Cas9/yw-gRNA (F_0 flies). Upper picture shows targeting site at *yellow* locus. Lower pictures show T7 endonuclease I (T7E1) assay of mutation induced by transgenic vasa-Cas9/U6B-y1-gRNA (left) and vasa-Cas9/CR7T-y2-gRNA (right). (B) Indel mutations induced by transgenic vasa-Cas9/pyw-gRNA at *yellow* locus. Representative DNA sequencing results of the PCR products from F_1 individual flies show indel mutations induced by transgenic vasa-Cas9/U6B-y1-gRNA (upper picture) and vasa-Cas9/U6B-y2-gRNA (lower picture) at the targeted locus. The wild-type DNA sequence is shown on the top with the target site underlined and the PAM sequence highlighted in red. Deletions are shown as red dashes and insertions highlighted in blue and lowercase letters. The change of DNA length (in nucleotides) caused by each mutation is indicated to the right of each sequence (+, insertion; -, deletion). Note that some alterations have both insertions and deletions of nucleotides and in these cases the alterations are enumerated in the brackets.

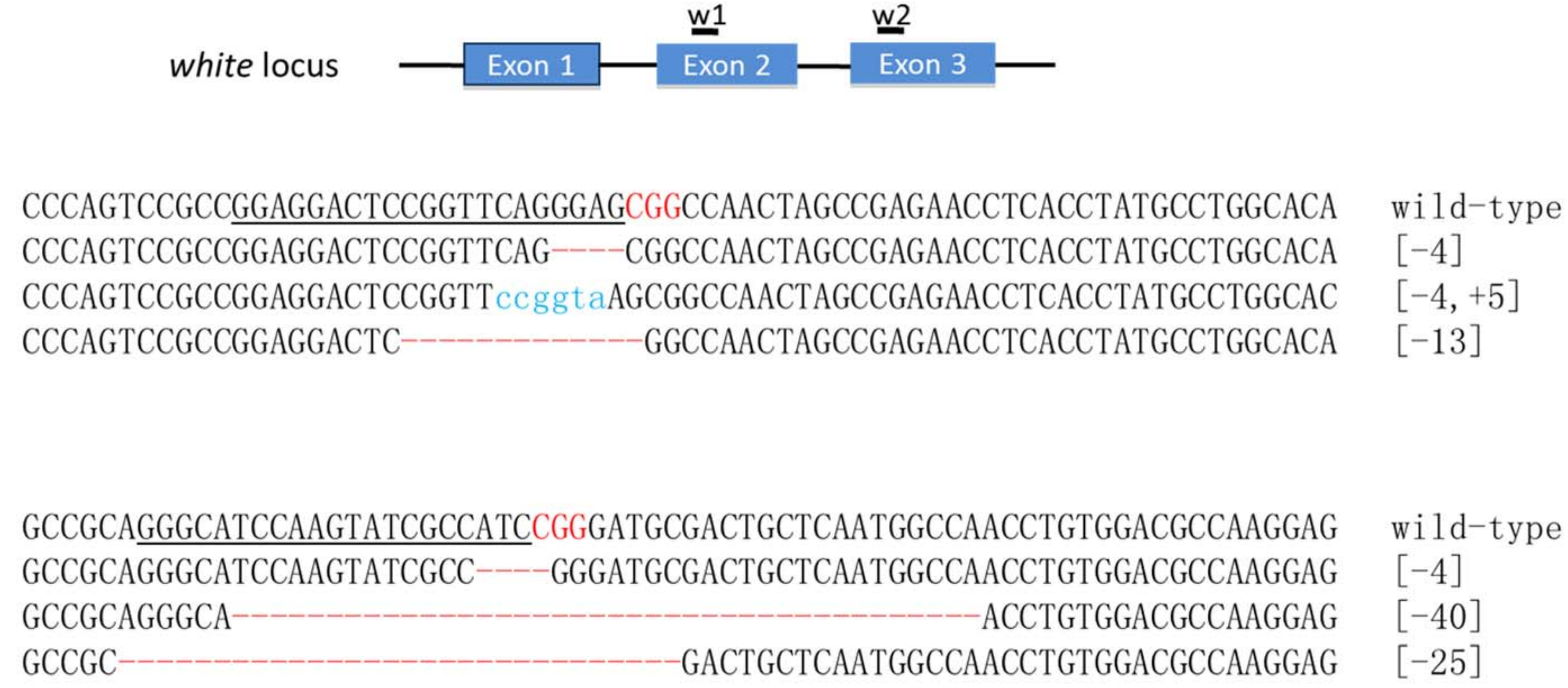

Figure S3 Indel mutations induced by transgenic vasa-Cas9/w-gRNA at *white* locus. Top picture shows two different targeting sites at *white* locus. Representative DNA sequencing results of the PCR products from F_1 individual flies show indel mutations induced by transgenic vasa-Cas9/CR7T-w1-gRNA (middle) and vasa-Cas9/CR7T-w2-gRNA (lower image) at the targeted *white* locus.

>Promoter of *U6B*
GTTCGACTTGCAGCCTGAAATACGGCACGAGTAGGAAAAGCCGAGTCAAATGCCGAATGCAGAGTCTCATTACAGCACAATCAACTC
AAGAAAAACTCGACACTTTTTACCATTTGCACTTAAATCCTTTTTATTCGTTATGTATACTTTTTTTGGTCCCTAACCAAAACAAAAC
CAAACTCTCTTAGTCGTGCCTCTATATTTAAAACTATCAATTTATTATAGTCAATAAATCGAACTGTGTTTTCAACAAACGAACAATAG
GACACTTTGATTCTAAAGGAAATTTTGAAAATCTTAAGCAGAGGGTTCTTAAGACCATTTGCCAATTCTTATAATTCTCAACTGCTCTT
TCCTGATGTTGATCATTTATATAGGTATGTTTTCCTCAATACTTC

>Promoter of *CR34335*
CCGTTTTGTCATCGCTTTTTGTCGGGTCTCAGTTGTGGATCGAAAACCCGGAGAGTAACCCCTTGACCCCTTGTCAGTTTCCGCATGCTC
CAGCCCTTGTCCTCATATTTTCCAATCGTTTTTGGCGTTTTTAGCTGCGGCCATTGAATGTTCAAAAATTATTGTAAACTTTTCAGACCC
CCTCCGTGTGTGTGTGTGTGTGTGCTTCTGTGTATGTGGAAAAGTTCGAATTCTAGTTGGGAAATCAATTAAAAGCGCTTGTTGGACTC
GCACCACAACTTTCCCTTTCGATCGCCCCTGCGGTTTGGTTCAACTGGTCAAGGCCTCTAGCTCCCAAAAACCGTCGAAAATTGGTTTG
GCACTTTTTATAGCCACCCAACATAATACGAAGTTCAGACAGAAATTGATATGGGTATTGGACTGATCAATAACTTAAGTTCCCTCACT
GAATTATCCTTAACTCTTGTGCATGCCTATTTATGTGAAAACAATCAAAATTACTCAATTATGTATGCACAGGTATTGAAAGCTCCCAC
TATTTAGTTTTTGTTTTAATTATCTGTCTTTAGAACTTTAAATGGAAAAACATGACTTATATGTATGAACAAAAACTCCAATCGATCAC
TTTAGGCGGCCGCCAATGCCAACAGAAGCATTATTTTTTTCCTTTCCCGATCTCTCAAGCACACTCAGTTGATCTCAATCGCAAAACTC
GTTACTCACTCTTAATATTTCCTCTTCTTTGCACTTTCCTGGTGAAATGCATTCTCTATTTTCCTCTCCCTACTGCACATACTCTTTGAGC
TCTCTAGCAGATACCCTAACAATTTCTAATCATCCACGAATAATAAACAAAGATTTCTAATATCTTAAACCTGTTTTATATACTTATTAT
TCAAAAAAAAAAAAGTGTGGCAAAAAAAAAGAGCACTTCAACGTTTGTTGAACAAAGGATATAATTTTGTAAGGGTATCTAGAAGTT
AAGTTTTCATGCCTCTCTTCATCCACTCTTCTCAACCTCATGCGATAGCTGCTGCGCTCTCTCGTTTATCGTTCGGTTGAGGTTTTATAAT
TCTCAAATACTTTTTCCCGAAGTGGTGCCTTTAAATAGCGTATATGAGTGGAAGACTTTCC

Figure S4 The DNA sequences of U6B promoter and CR34335 promoter used in our study.

>attP-FRT-RFP
CGCGCTCGCGCGACTGACGGTCGTAAGCACCCGCGTACGTGTCCACCCCGGTCACAACCCCTTGTGTCATGTCGGCGGCCCTACGCCC
CCAACTGAGAGAACTCAAAGGTTACCCCAGTTGGGGCACTACTCCCGAAAACCGCTTCTGACCTGGGAAAACGTGAAGCCCCGGGGC
ATCCGCTGAGGGTTGCCGCCGGGGCTTCGGTGTGTCCGTCAGTACGAAGTTCCTATTCTCTAGAAAGTATAGGAACTTCGGATCTAATT
CAATTAGAGACTAATTCAATTAGAGCTAATTCAATTAGGATCCAAGCTTATCGATTTCGAACCCTCGACCGCCGGAGTATAAATAGAG
GCGCTTCGTCTACGGAGCGACAATTCAATTCAAACAAGCAAAGTGAACACGTCGCTAAGCGAAAGCTAAGCAAATAAACAAGCGCAG
CTGAACAAGCTAAACAATCGGGACTAGAGCCGGTCGCCACCATGAGGTCTTCCAAGAATGTTATCAAGGAGTTCATGAGGTTTAAGGT
TCGCATGGAAGGAACGGTCAATGGGCACGAGTTTGAAATAGAAGGCGAAGGAGAGGGGAGGCCATACGAAGGCCACAATACCGTAA
AGCTTAAGGTAACCAAGGGGGGACCTTTGCCATTTGCTTGGGATATTTTGTCACCACAATTTCAGTATGGAAGCAAGGTATATGTCAA
GCACCCTGCCGACATACCAGACTATAAAAAGCTGTCATTTCCTGAAGGATTTAAATGGGAAAGGGTCATGAACTTTGAAGACGGTGGC
GTCGTTACTGTAACCCAGGATTCCAGTTTGCAGGATGGCTGTTTCATCTACAAGGTCAAGTTCATTGGCGTGAACTTTCCTTCCGATGG
ACCTGTTATGCAAAAGAAGACAATGGGCTGGGAAGCCAGCACTGAGCGTTTGTATCCTCGTGATGGCGTGTTGAAAGGAGAGATTCA
TAAGGCTCTGAAGCTGAAAGACGGTGGTCATTACCTAGTTGAATTCAAAAGTATTTACATGGCAAAGAAGCCTGTGCAGCTACCAGG
GTACTACTATGTTGACTCCAAACTGGATATAACAAGCCACAACGAAGACTATACAATCGTTGAGCAGTATGAAAGAACCGAGGGACG
CCACCATCTGTTCCTTTAGCGGCCATCGAATTCGAGCTCGCCCACTAAGCGTCGCGCCACTTCAACGCTCGATGGGAGCGTCATTGGTG
GGCGGGGTAACCGTCGAAATCAGTGTTTACGCTTCCAATCGCAACAAAAAATTCACTGCAACACTGAAAAGCATACGAAAACGATGA
AGATTGTACGAGAAACCATAAAGTATTTTATCCACAAAGACACGTATAGCAGAAAAGCCAAGTTAACTCGGCGATAAGTTGTGTACA
CAAGAATAAAATCGGCCAGATTCAGTGTTGTCAGAAATAAGAAAACCCCACTATGTTTTTCTTTGCCTTTTCTTTCTCCCAGCGATCAT
TCATTTCGTGGTGAAAGAACGGGGTCATTGCACGGAGTTTCGACTGCGGGAAAGCAGAGCTGCCGTTCACTTCGTCTATAATTAGCGC
TTTCTATTTTCCCCGATTCGGGCCGCTGCTGCGCTTTTCCGCCTGCTGTTTGTGGCAAGTGTAGCAGCAGGCTGTGCACGCAGTGTGGC
ATGCACTTGGCTTTCCACCGTTGGTATCGATTCTCTGGGACGATGAGTCATTCCTTTCGGGGCCACAGCATAATCGTTGCCAGCTCACC
GAAATGGTGACTTCATTTCTTAACTGCCGTCAAGCATGCGATTGTACATACATACATATTTATATATGTACATATTTATGTGACTATGG
TAGGTCGATATAATAGCAATCAACGCAAGCAAATGTGTCAGTCCTGCTTACAGGAACGATTCTATTTAGTAATTTTCGTTGTATAAAGT
AATTATGTATGTATGTAAGCCCCATAAATCTGAAACAATTAGGCAAAACCATGCGAAGCT

Figure S5 The DNA sequence of the attP-FRT-RFP cassette in our study. The attP sequence is highlighted in red and the FRT highlighted in green, and the RFP DNA sequence is shown as underlined.

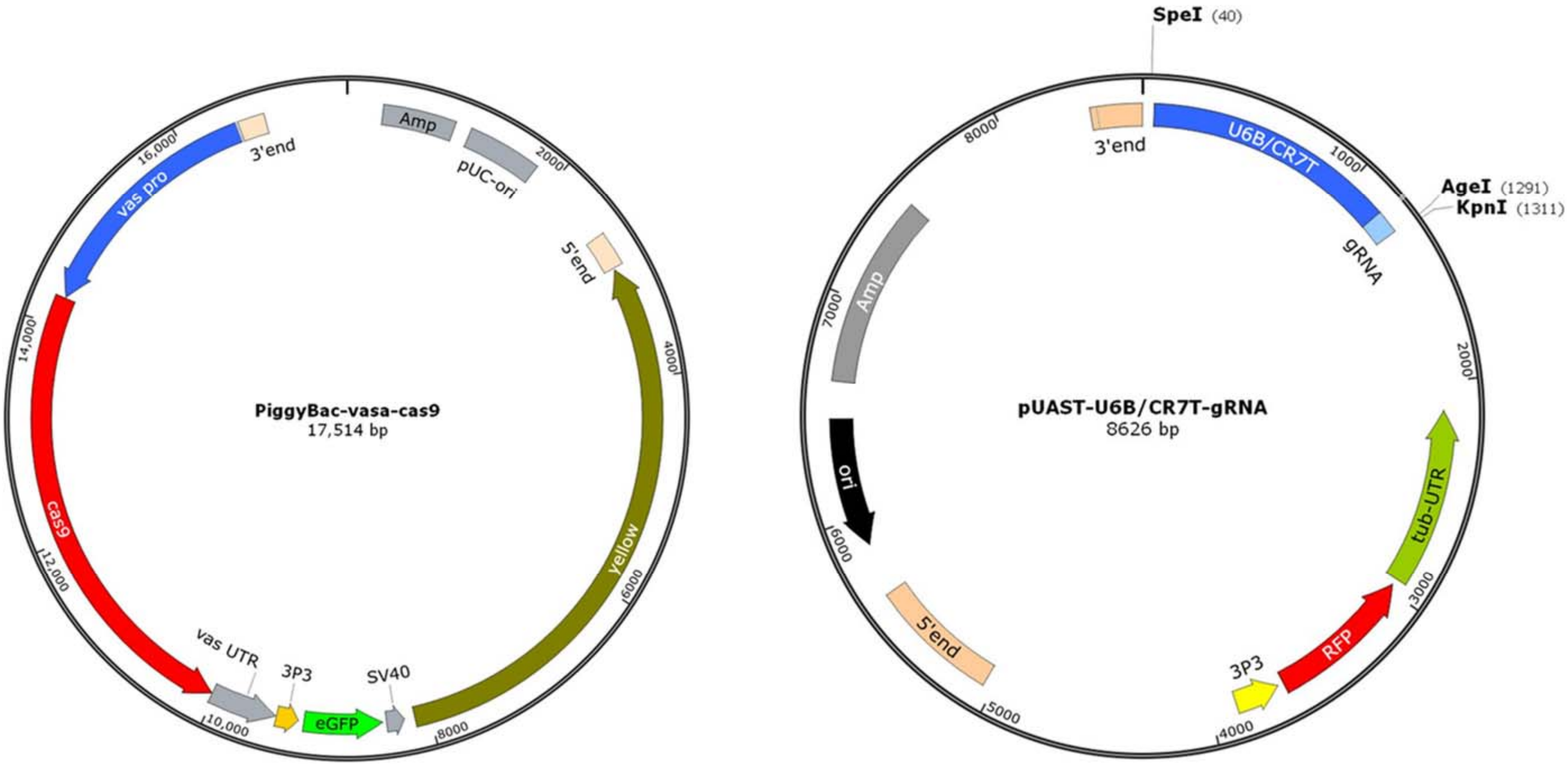

Figure S6 Maps of the plasmids with vasa-cas9 or the U6B/CR7T promoters.

Table S1 ***Drosophila*** **gene sites targeted in this study**

**Target gene**		**Target site (5' to 3') (PAM is underlined)**
ms(3)K81		GGATTTCTGATTACGCGGTACGG
yellow	*y1*	GGATGAGTGTGGTCGGCTGTGGG
	y2	GGGTTTTGGACACTGGAACCGTGG
white	*w1*	GGAGGACTCCGGTTCAGGGAGCGG
	w2	GGGCATCCAAGTATCGCCATCCGG
Hisc-RA		GGACTTACAGCTGTACGTTGTGG

Table S2-I List of primers for vasa-Cas9/pUAST-gRNA vector constructions used in this study

Plasmid	Primer name	Primer sequence (5' – 3') Forward and Reverse
piggyBac-vasa-cas9	Vaspro-F	CCCGGGTACCTGCAGCTGGTTGTAGGTGCAGTTG
	Vaspro-R	GGCGCGCCTAGAGACTAGTGCGGCCGCATTGATATTTTTTTTTTAATTTGGCCTGC
	VasUTR-F	ACTAGTCTCGAGAATGTATGGACATAGATTTCAAATAATTAAATG
	VasUTR-R	GGCGCGCCAACACGAAGAGCAGCAGTGTGGT
	PigGFP-F	CTACCCGGGACTGATACTAGTATCTAATTCAATTAGAGACTAATTCAAT
	PigGFP-R	CTAGGGCCCGTACGCGTATCGATAAGCTTTAA
	PigGFP-KOD-F	ACCGCGGGCGCGGGATCCACCGGTCGCCACC
	PigGFP-KOD-R	ACCGTCGACTCTAGCGGTACC
pUAST-U6B/CRT-gRNA	Pro-U6B400-NotsphspeFse-F	GCGGCCGCATGCACTAGTGGCCGGCCGTTCGACTTGCAGCCTGAAATAC
	Pro-U6B400-AscI-R1	GGCGCGCCGAAGTATTGAGGAAAACATACCTATATA
	Pro-U6B400-AscI-R1	GGCGCGCCGAAGTATTGAGGAAAACATACCTATATA
	Pro-U6A100-Not-F	GCGGCCGCAGACACAGCGCGTACGTCCTTC
	Pro-U6A100-Asc-R1	CGGACTAGCCTTATTTTAACTTGCTATTTCTAGCTCTAAAACGGCGCGCCGAAGTTCACCCGGATATCTTTC
	Pro-U6A100-Acc65-R2	GGTACCAAAAAAAGCACCGACTCGGTGCCACTTTTTCAAGTTGATAACGGACTAGCCTTATTTTAACTTG
	Pro-CR34335-XhospeFse-F	CTCGAGACTAGTGGCCGGCCCGTTTTGTCATCGCTTTTTGTCG
	Pro-CR34335-Asc-R	GGCGCGCCGAAAGTCTTCCACTCATATACGCTA
	gRNA-AscI-F	GGCGCGCCGTTTTAGAGCTAGAAATAGC
	6B/CR34335-Age/Kpn-R	GGTACCTGTTTAAACTACCGGTAAAAAAAGCACCGACTCGGTGCCAC

pUAST-U6B/CRT-gRNA (II)	U6-Age-F	CATACCGGTGTTCGACTTGCAGCCTGAAATAC
	CRU6/34335II-Kpn-R	CATGGTACCAAAAAAAGCACCGACTCGGTGCCAC
	CR34335II-Age-F	CATACCGGTCGTTTTGTCATCGCTTTTTGTCG
	CRU6/34335II-Kpn-R	CATGGTACCAAAAAAAGCACCGACTCGGTGCCAC

Table S2-II List of primers for transgenic gRNA vectors constructions used in this study

Target locus	Primer name	Primer sequence (5' – 3') Forward and Reverse
ms(3)k81	K81-KOD-F	TTACGCGGTAGTTTTAGAGCTAGAAATAGCAAGTT
	K81-KOD-R	TCAGAAATCCGAAGTATTGAGGAAAACATACCTA
yellow	yw-gRNA-KOD-F	GGTCGGCTGTGTTTTAGAGCTAGAAATAGCAAGTT
	yw-gRNA-KOD-R	ACACTCATCCGAAGTATTGAGGAAAACATACCTA
white	CR-W1-KOD-R	CTCCCTGAACCGGAGTCCTCCGAAAGTCTTCCACTCATATACGCTA
	CRW4-KOD-R	GATGGCGATACTTGGATGCCCGAAAGTCTTCCACTCATATACGCTA
	gRNA-KOD-F	GTTTTAGAGCTAGAAATAGCAAGTT
pUAST-3p3-RFP	3P3RPF-EcoRVF	CATGATATCCCGGGGATCTAATTCAATTAG
	3P3RPF-EcoRVR	CATGATATCGAGCTTCGCATGGTTTTGCC

Table S3 List of primers for PCR check in mutations used in this study

Target locus	Primer name	Primer sequence (5' – 3') Forward and Reverse
yellow	yellow-F	CGGAGCTAATTCCGTATCCA
	yellow-R	CGCCAGGTAGCTCGTATCTC
ms(3)k81	CG14251 -F	GAGATTTCTCACTACTGCTCCTCG
	CG14251 -R	ACACGAATTGGATATGCGATAGC
white	White-Seq-F1	GGTTAGATGAGCATAACGCTTGTAG
	White-Seq-R1	CCACGCTGGATAGGAGTTGAGAT
Hisc-RA	Hisc-RA-HLF	CTAACCGGTTAGGGAGTTAGAGTGGTCGTGGC
	Hisc-RA-HLR	CAGGCGGCCGCCGTACAGCTGTAAGTCCTTGCTGA
	Hisc-RA-HRF	GGCGCGCCTTGTGGCATAGTATGAGCGATTGC
	Hisc-RA-HRR	ACTAGTTAGTTCGTATCAACACTCTACCCCAG
	Hisc-RA-F01	TTGTAAACCCAACTATCCTATCCG
	Hisc-RA-R01	CCAAGCAAATGGCAAAGGTCC
	Hisc-RA-F02	TGATGGCGTGTTGAAAGGAGAGA
	Hssc-RA-R02	GCAACTAGTGCTCTTAGCACTTTCTTG
	Hisc-RA-F03	TACGAGGAAGAATGAGACAACCA
	Hisc-RA-R03	TATAAGGACGGCACCAAAGCGC
