# Supplementary material for: Efficient Gene Knock-out and Knock-in with Transgenic Cas9 in Drosophila
Source: G3 (Bethesda). 2014 Mar 21;4(5):925–9. doi: 10.1534/g3.114.010496 (PMC4025491; doi:10.1534/g3.114.010496)
Supplement: Supporting Information [file supp_g3.114.010496_FigureS1.pdf]

A

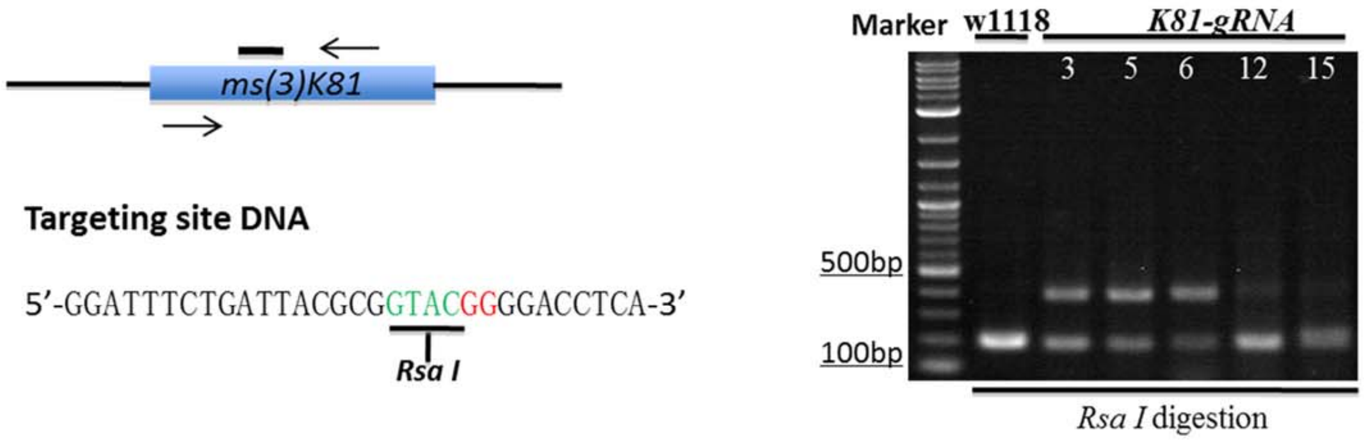

AACTTCAGCAGGTGCGGATTTCTGATTACGCGGTACGGGGACCTCAGCGTGTGATTCCCC wild-type
AACTTCAGCAGGTGCGGATTTCTGATTACGCGgTACGGGGACCTCAGCGTGTGATTCCCC [+1]
AACTTCAGCAGGTGCGGATTTCTGATTACGCG-TACGGGGACCTCAGCGTGTGATTCCCC [-1]
AACTTCAGCAGGTGCGGATTTCTGATTACGCGGTACGGGGACCgggaccTCAGCGTGTGA [+6]

B

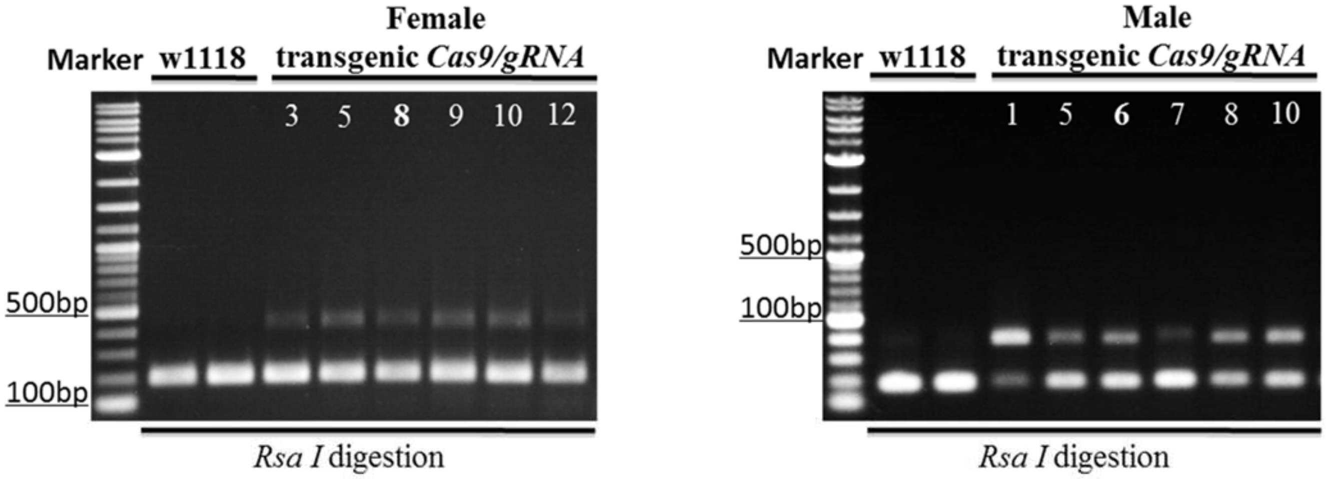


```
AACTTCAGCAGGTGCGGATTTCTGATTACGCGGTACGGGGACCTCAGCGTGTGATTCCCC   wild-type
AACTTCAGCAGGTGCGGATTTCTGATTACGCG--------ACCTCAGCGTGTGATTCCCC   [-8]
AACTTCAGCAGGTGCGGATTTC----------GTACGGGGACCTCAGCGTGTGATTCCCC   [-10]
AACTTCAGCAGGTGCGGATTTCTGATTACG-------------------TGTGATTCCCC   [-19]
AACTTCAGCAGGTGCGGATTTCTGATTACG--GTACGGGGACCTCAGCGTGTGATTCCCC   [-2]
```

Figure S1 (A) Mutations induced by injection of k81-gRNA in transgenic vasa-Cas9 embryos. Images on the top left corner of each panel show targeting site. Enzyme cutting sites chosen for identification of mutations are underlined. Images on each top right corner show the enzyme digestion results of PCR products of wild-type and transgenic Cas9/gRNA-induced F_0 mutants. Representative DNA sequencing results of the PCR products from F_1 individual flies show indel mutations induced by transgenic Cas9/gRNA at the targeted *ms(3)k81* locus. The wild-type DNA sequence is shown on the top with the target site underlined and the PAM sequence highlighted in red. Deletions are shown as red dashes and insertions highlighted in blue and lowercase letters. The change of DNA length (in nucleotides) in each mutation is indicated to the right of each sequence (+, insertion; -, deletion). (B) Mutations induced by transgenic vasa-Cas9/k81-gRNA at *ms(3)k81*. Upper pictures show the enzyme digestion results of PCR products of wild-type and transgenic Cas9/gRNA-induced F_0 mutants. Lower pictures show representative DNA sequencing results of the PCR products from F_1 individual flies showing indel mutations induced by transgenic Cas9/gRNA at the targeted *ms(3)k81* locus.
