# Supplementary material for: Efficient Gene Knock-out and Knock-in with Transgenic Cas9 in Drosophila
Source: G3 (Bethesda). 2014 Mar 21;4(5):925–9. doi: 10.1534/g3.114.010496 (PMC4025491; doi:10.1534/g3.114.010496)
Supplement: Supporting Information [file supp_g3.114.010496_FigureS2.pdf]

A

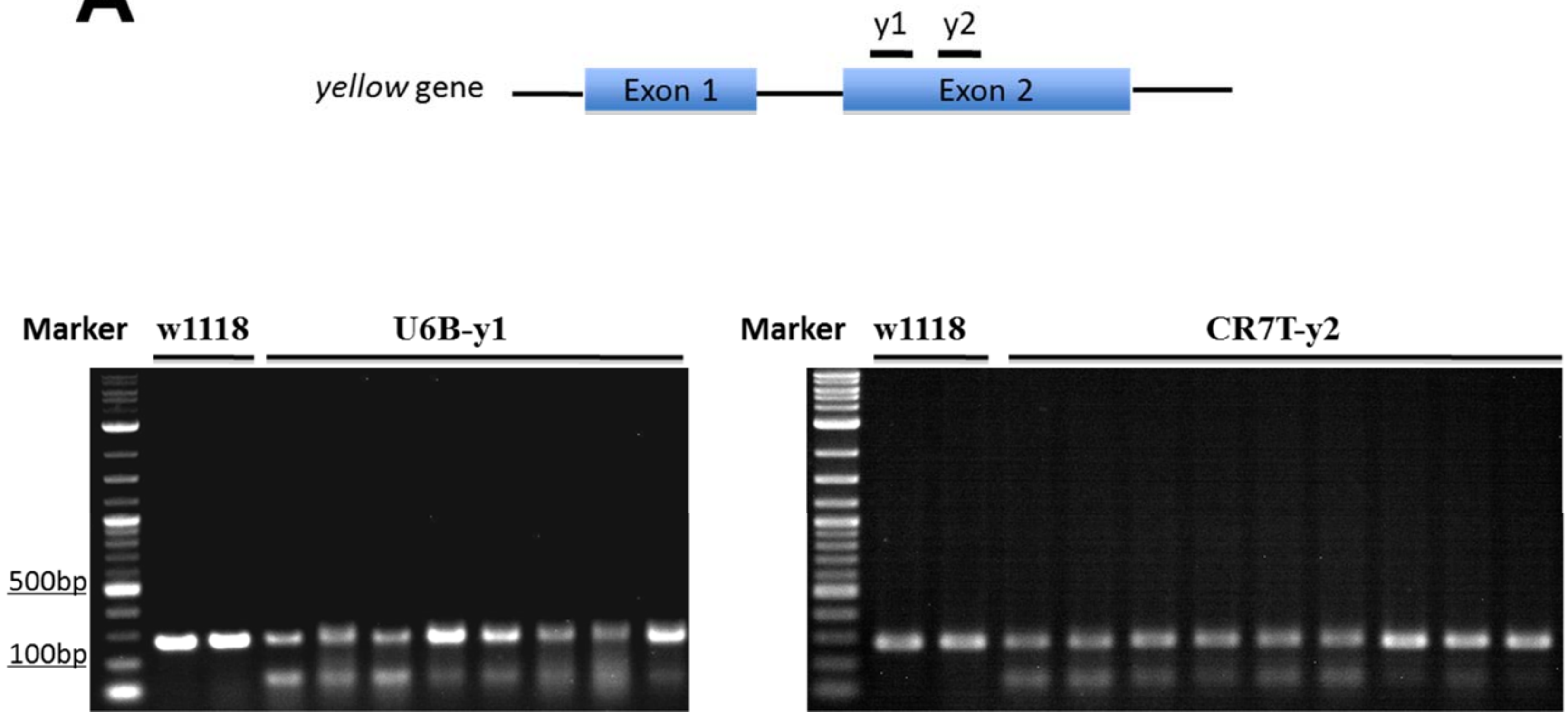
y1
y2
yellow gene
Exon 1
Exon 2
Marker
w1118
U6B-y1
500bp
100bp
Marker
w1118
CR7T-y2

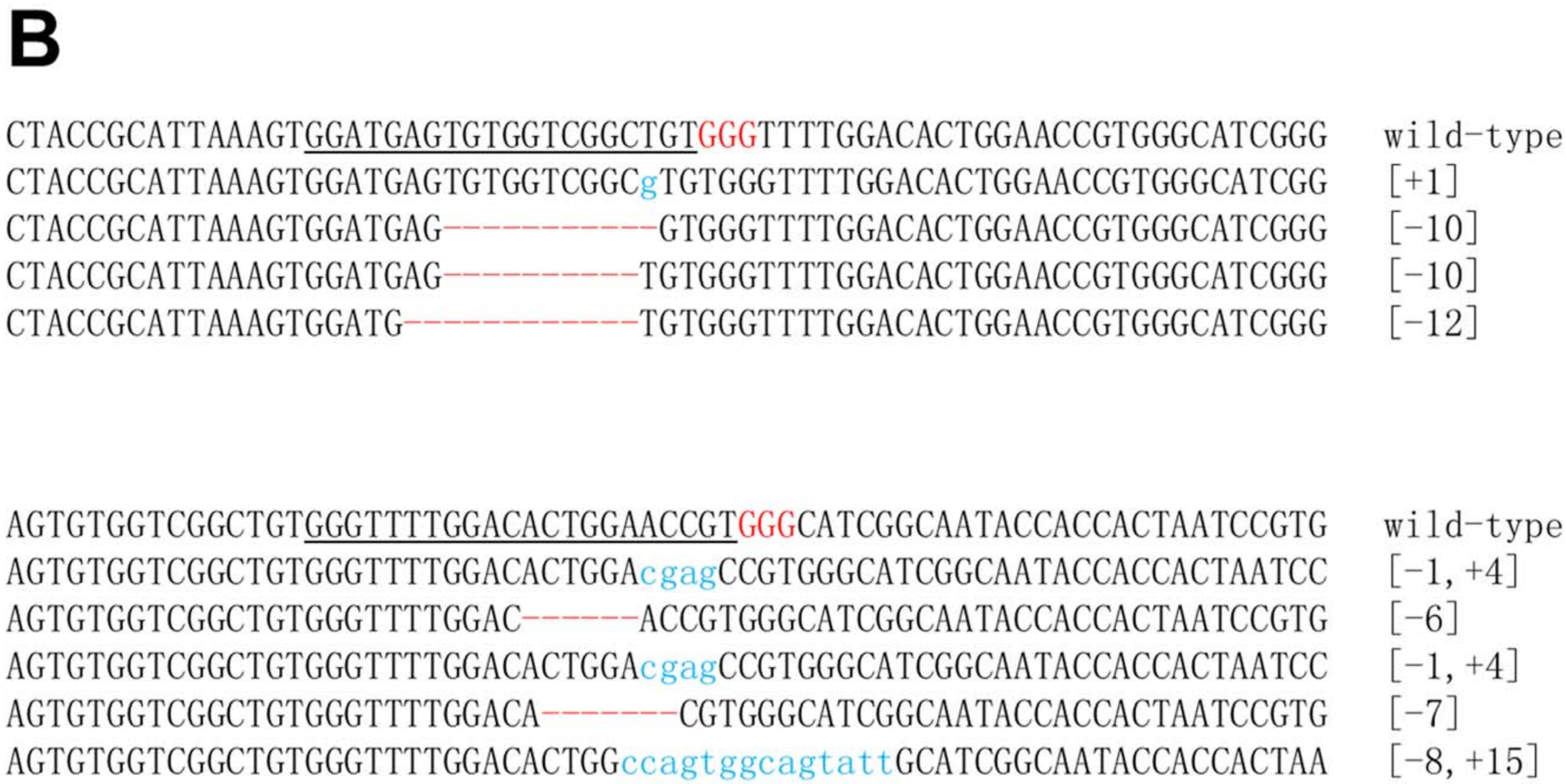


**Figure S2** (A) T7 endonuclease I (T7E1) assay of mutation at *yellow* locus induced by transgenic vasa-Cas9/yw-gRNA ($F_0$ flies). Upper picture shows targeting site at *yellow* locus. Lower pictures show T7 endonuclease I (T7E1) assay of mutation induced by transgenic vasa-Cas9/U6B-y1-gRNA (left) and vasa-Cas9/CR7T-y2-gRNA (right). (B) Indel mutations induced by transgenic vasa-Cas9/pyw-gRNA at *yellow* locus. Representative DNA sequencing results of the PCR products from $F_1$ individual flies show indel mutations induced by transgenic vasa-Cas9/U6B-y1-gRNA (upper picture) and vasa-Cas9/U6B-y2-gRNA (lower picture) at the targeted locus. The wild-type DNA sequence is shown on the top with the target site underlined and the PAM sequence highlighted in red. Deletions are shown as red dashes and insertions highlighted in blue and lowercase letters. The change of DNA length (in nucleotides) caused by each mutation is indicated to the right of each sequence (+, insertion; -, deletion). Note that some alterations have both insertions and deletions of nucleotides and in these cases the alterations are enumerated in the brackets.
