# Supplementary material for: Efficient Gene Knock-out and Knock-in with Transgenic Cas9 in Drosophila
Source: G3 (Bethesda). 2014 Mar 21;4(5):925–9. doi: 10.1534/g3.114.010496 (PMC4025491; doi:10.1534/g3.114.010496)
Supplement: Supporting Information [file supp_g3.114.010496_FigureS3.pdf]

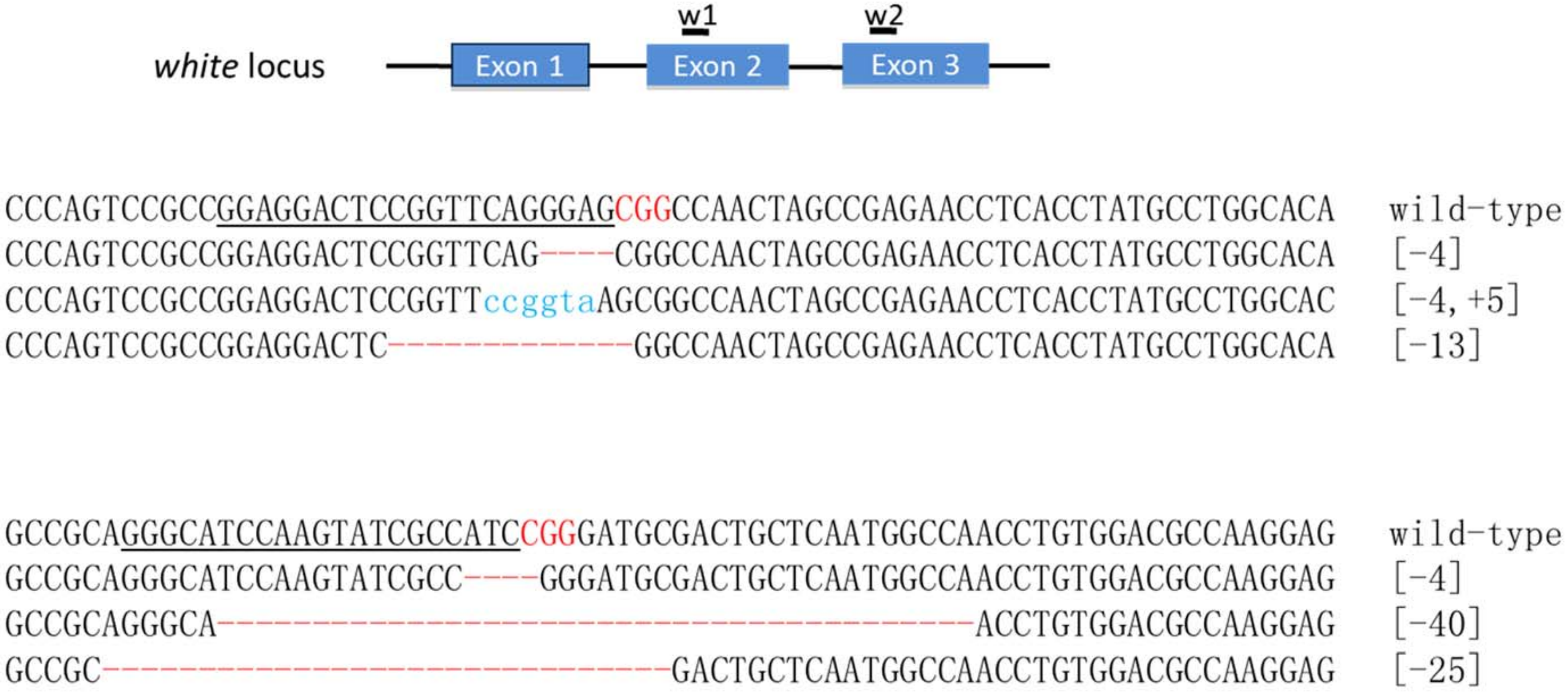


**Figure S3** Indel mutations induced by transgenic vasa-Cas9/w-gRNA at *white* locus. Top picture shows two different targeting sites at *white* locus. Representative DNA sequencing results of the PCR products from $F_1$ individual flies show indel mutations induced by transgenic vasa-Cas9/CR7T-w1-gRNA (middle) and vasa-Cas9/CR7T-w2-gRNA (lower image) at the targeted *white* locus.
