# Supplementary material for: Efficient Gene Knock-out and Knock-in with Transgenic Cas9 in Drosophila
Source: G3 (Bethesda). 2014 Mar 21;4(5):925–9. doi: 10.1534/g3.114.010496 (PMC4025491; doi:10.1534/g3.114.010496)
Supplement: Supporting Information [file supp_g3.114.010496_FigureS4.pdf]

>Promoter of *U6B*
GTTCGACTTGCAGCCTGAAATACGGCACGAGTAGGAAAAGCCGAGTCAAATGCCGAATGCAGAGTCTCATTACAGCACAATCAACTC
AAGAAAAACTCGACACTTTTTTACCATTTGCACTTAAATCCTTTTTTATTCGTTATGTATACTTTTTTTGGTCCCTAACCAAAACAAAAC
CAAACTCTCTTAGTCGTGCCTCTATATTTAAAACTATCAATTTATTATAGTCAATAAATCGAACTGTGTTTTCAACAAACGAACAATAG
GACACTTTGATTCTAAAGGAAATTTTGAAAATCTTAAGCAGAGGGTTCTTAAGACCATTTGCCAATTCTTATAATTCTCAACTGCTCTT
TCCTGATGTTGATCATTTATATAGGTATGTTTTCCTCAATACTTC

>Promoter of *CR34335*
CCGTTTTGTCATCGCTTTTTGTCGGGTCTCAGTTGTGGATCGAAAACCCGGAGAGTAACCCCTTGACCCCTTGTCAGTTTCCGCATGCTC
CAGCCCTTGTCCTCATATTTTCCAATCGTTTTTGGCGTTTTTAGCTGCGGCCATTGAATGTTCAAAAATTATTGTAAACTTTTCAGACCC
CCTCCGTGTGTGTGTGTGTGTGTGCTTCTGTGTATGTGGAAAAGTTCGAATTCTAGTTGGGAAATCAATTAAAAGCGCTTGTTGGACTC
GCACCACAACTTTCCCTTTCGATCGCCCCTGCGGTTTGGTTCAACTGGTCAAGGCCTCTAGCTCCCAAAAACCGTCGAAAATTGGTTTG
GCACTTTTTATAGCCACCCAACATAATACGAAGTTCAGACAGAAATTGATATGGGTATTGGACTGATCAATAACTTAAGTTCCCTCACT
GAATTATCCTTAACTCTTGTGCATGCCTATTTATGTGAAAACAATCAAAATTACTCAATTATGTATGCACAGGTATTGAAAGCTCCCAC
TATTTAGTTTTTGTTTTAATTATCTGTCTTTAGAACTTTAAATGGAAAAACATGACTTATATGTATGAACAAAAACTCCAATCGATCAC
TTTAGGCGGCCGCCAATGCCAACAGAAGCATTATTTTTTTCCTTTCCCGATCTCTCAAGCACACTCAGTTGATCTCAATCGCAAAACTC
GTTACTCACTCTTAATATTTCCTCTTCTTTGCACTTTCCTGGTGAAATGCATTCTCTATTTTCCTCTCCCTACTGCACATACTCTTTGAGC
TCTCTAGCAGATACCCTAACAATTTCTAATCATCCACGAATAATAAACAAAGATTTCTAATATCTTAAACCTGTTTTATATACTTATTAT
TCAAAAAAAAAAAAGTGTGGCAAAAAAAAAAGAGCACTTCAACGTTTGTTGAACAAAGGATATAATTTTGTAAGGGTATCTAGAAGTT
AAGTTTTCATGCCTCTCTTCATCCACTCTTCTCAACCTCATGCGATAGCTGCTGCGCTCTCTCGTTTATCGTTCGGTTGAGGTTTTATAAT
TCTCAAATACTTTTTCCCGAAGTGGTGCCTTTAAATAGCGTATATGAGTGGAAGACTTTCC

Figure S4 The DNA sequences of U6B promoter and CR34335 promoter used in our study.
