# Supplementary material for: Efficient Gene Knock-out and Knock-in with Transgenic Cas9 in Drosophila
Source: G3 (Bethesda). 2014 Mar 21;4(5):925–9. doi: 10.1534/g3.114.010496 (PMC4025491; doi:10.1534/g3.114.010496)
Supplement: Supporting Information [file supp_g3.114.010496_FigureS5.pdf]

>attP-FRT-RFP

CGCGCTCGCGCGACTGACGGTCGTAAGCACCCGCGTACGTGTCCACCCCGGTCACAACCCCTTGTGTCATGTCGGCGGCCCTACGCCC
CCAACTGAGAGAACTCAAAGGTTACCCCAGTTGGGGCACTACTCCCGAAAACCGCTTCTGACCTGGGAAAACGTGAAGCCCCGGGGC
ATCCGCTGAGGGTTGCCGCCGGGGCTTCGGTGTGTCCGTCAGTACGAAGTTCCTATTCTCTAGAAAGTATAGGAACTTCGGATCTAATT
CAATTAGAGACTAATTCAATTAGAGCTAATTCAATTAGGATCCAAGCTTATCGATTTCGAACCCTCGACCGCCGGAGTATAAATAGAG
GCGCTTCGTCTACGGAGCGACAATTCAATTCAAACAAGCAAAGTGAACACGTCGCTAAGCGAAAGCTAAGCAAATAAACAAGCGCAG
CTGAACAAGCTAAACAATCGGGACTAGAGCCGGTCGCCACCATGAGGTCTTCCAAGAATGTTATCAAGGAGTTCATGAGGTTTAAGGT
TCGCATGGAAGGAACGGTCAATGGGCACGAGTTTGAAATAGAAGGCGAAGGAGAGGGGAGGCCATACGAAGGCCACAATACCGTAA
AGCTTAAGGTAACCAAGGGGGGACCTTTGCCATTTGCTTGGGATATTTTGTCACCACAATTTCAGTATGGAAGCAAGGTATATGTCAA
GCACCCTGCCGACATACCAGACTATAAAAAGCTGTCATTTCCTGAAGGATTTAAATGGGAAAGGGTCATGAACTTTGAAGACGGTGGC
GTCGTTACTGTAACCCAGGATTCCAGTTTGCAGGATGGCTGTTTCATCTACAAGGTCAAGTTCATTGGCGTGAACTTTCCTTCCGATGG
ACCTGTTATGCAAAAGAAGACAATGGGCTGGGAAGCCAGCACTGAGCGTTTGTATCCTCGTGATGGCGTGTTGAAAGGAGAGATTCA
TAAGGCTCTGAAGCTGAAAGACGGTGGTCATTACCTAGTTGAATTCAAAAGTATTTACATGGCAAAGAAGCCTGTGCAGCTACCAGG
GTACTACTATGTTGACTCCAAACTGGATATAACAAGCCACAACGAAGACTATACAATCGTTGAGCAGTATGAAAGAACCGAGGGACG
CCACCATCTGTTCCTTTAGCGGCCATCGAATTCGAGCTCGCCCACTAAGCGTCGCGCCACTTCAACGCTCGATGGGAGCGTCATTGGTG
GGCGGGGTAACCGTCGAAATCAGTGTTTACGCTTCCAATCGCAACAAAAAATTCACTGCAACACTGAAAAGCATACGAAAACGATGA
AGATTGTACGAGAAACCATAAAGTATTTTATCCACAAAGACACGTATAGCAGAAAAGCCAAGTTAACTCGGCGATAAGTTGTGTACA
CAAGAATAAAATCGGCCAGATTCAGTGTTGTCAGAAATAAGAAAACCCCACTATGTTTTTCTTTGCCTTTTCTTTCTCCCAGCGATCAT
TCATTTCGTGGTGAAAGAACGGGGTCATTGCACGGAGTTTCGACTGCGGGAAAGCAGAGCTGCCGTTCACTTCGTCTATAATTAGCGC
TTTCTATTTTCCCCGATTCGGGCCGCTGCTGCGCTTTTCCGCCTGCTGTTTGTGGCAAGTGTAGCAGCAGGCTGTGCACGCAGTGTGGC
ATGCACTTGGCTTTCCACCGTTGGTATCGATTCTCTGGGACGATGAGTCATTCCTTTCGGGGCCACAGCATAATCGTTGCCAGCTCACC
GAAATGGTGACTTCATTTCTTAACTGCCGTCAAGCATGCGATTGTACATACATACATATTTATATATGTACATATTTATGTGACTATGG
TAGGTCGATATAATAGCAATCAACGCAAGCAAATGTGTCAGTCCTGCTTACAGGAACGATTCTATTTAGTAATTTTCGTTGTATAAAGT
AATTATGTATGTATGTAAGCCCCATAAATCTGAAACAATTAGGCAAAACCATGCGAAGCT

Figure S5 The DNA sequence of the attP-FRT-RFP cassette in our study. The attP sequence is highlighted in red and the FRT highlighted in green, and the RFP DNA sequence is shown as underlined.
