# Supplementary figures and images for: Efficient Gene Knock-out and Knock-in with Transgenic Cas9 in Drosophila
Source: G3 (Bethesda). 2014 Mar 21;4(5):925–9. doi: 10.1534/g3.114.010496 (PMC4025491; doi:10.1534/g3.114.010496)

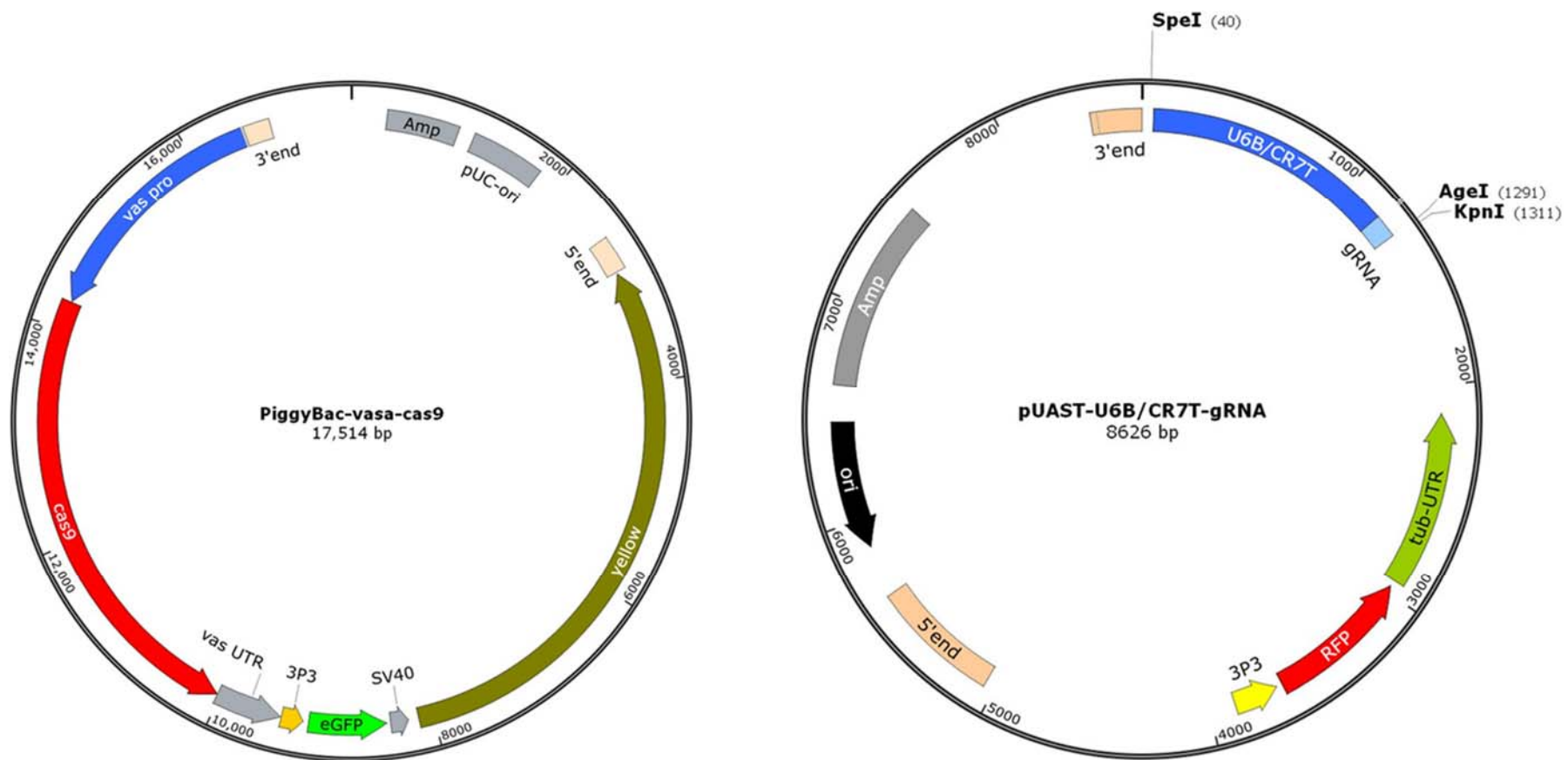

**Figure S6** Maps of the plasmids with vasa-cas9 or the U6B/CR7T promoters.

Supplement: Supporting Information [file supp_g3.114.010496_FigureS6.pdf]
