# Supplementary material for: Efficient Gene Knock-out and Knock-in with Transgenic Cas9 in Drosophila
Source: G3 (Bethesda). 2014 Mar 21;4(5):925–9. doi: 10.1534/g3.114.010496 (PMC4025491; doi:10.1534/g3.114.010496)
Supplement: Supporting Information [file supp_g3.114.010496_TableS1.pdf]

Table S1 ***Drosophila*** **gene sites targeted in this study**

**Target gene**		**Target site (5' to 3') (PAM is underlined)**
ms(3)K81		GGATTTCTGATTACGCGGTACGG
yellow	*y1*	GGATGAGTGTGGTCGGCTGTGGG
	y2	GGGTTTTGGACACTGGAACCGTGG
white	*w1*	GGAGGACTCCGGTTCAGGGAGCGG
	w2	GGGCATCCAAGTATCGCCATCCGG
Hisc-RA		GGACTTACAGCTGTACGTTGTGG
