# Supplementary material for: Efficient Gene Knock-out and Knock-in with Transgenic Cas9 in Drosophila
Source: G3 (Bethesda). 2014 Mar 21;4(5):925–9. doi: 10.1534/g3.114.010496 (PMC4025491; doi:10.1534/g3.114.010496)
Supplement: Supporting Information [file supp_g3.114.010496_TableS2.pdf]

Table S2-I List of primers for vasa-Cas9/pUAST-gRNA vector constructions used in this study

Plasmid	Primer name	Primer sequence (5' – 3') Forward and Reverse
piggyBac-vasa-cas9	Vaspro-F	CCCGGGTACCTGCAGCTGGTTGTAGGTGCAGTTG
	Vaspro-R	GGCGCGCCTAGAGACTAGTGCGGCCGCATTGATATTTTTTTTTTAATTTGGCCTGC
	VasUTR-F	ACTAGTCTCGAGAATGTATGGACATAGATTTCAAATAATTAAATG
	VasUTR-R	GGCGCGCCAACACGAAGAGCAGCAGTGTGGT
	PigGFP-F	CTACCCGGGACTGATACTAGTATCTAATTCAATTAGAGACTAATTCAAT
	PigGFP-R	CTAGGGCCCGTACGCGTATCGATAAGCTTTAA
	PigGFP-KOD-F	ACCGCGGGCGCGGGATCCACCGGTCGCCACC
	PigGFP-KOD-R	ACCGTCGACTCTAGCGGTACC
pUAST-U6B/CRT-gRNA	Pro-U6B400-NotsphspeFse-F	GCGGCCGCATGCACTAGTGGCCGGCCGTTCGACTTGCAGCCTGAAATAC
	Pro-U6B400-AscI-R1	GGCGCGCCGAAGTATTGAGGAAAACATACCTATATA
	Pro-U6B400-AscI-R1	GGCGCGCCGAAGTATTGAGGAAAACATACCTATATA
	Pro-U6A100-Not-F	GCGGCCGCAGACACAGCGCGTACGTCCTTC
	Pro-U6A100-Asc-R1	CGGACTAGCCTTATTTTAACTTGCTATTTCTAGCTCTAAAACGGCGCGCCGAAGTTCACCCGGATATCTTTC
	Pro-U6A100-Acc65-R2	GGTACCAAAAAAAGCACCGACTCGGTGCCACTTTTTCAAGTTGATAACGGACTAGCCTTATTTTAACTTG
	Pro-CR34335-XhospeFse-F	CTCGAGACTAGTGGCCGGCCCGTTTTGTCATCGCTTTTTGTCG
	Pro-CR34335-Asc-R	GGCGCGCCGAAAGTCTTCCACTCATATACGCTA
	gRNA-AscI-F	GGCGCGCCGTTTTAGAGCTAGAAATAGC
	6B/CR34335-Age/Kpn-R	GGTACCTGTTTAAACTACCGGTAAAAAAAGCACCGACTCGGTGCCAC

pUAST-U6B/CRT-gRNA (II)	U6-Age-F	CATACCGGTGTTCGACTTGCAGCCTGAAATAC
	CRU6/34335II-Kpn-R	CATGGTACCAAAAAAAGCACCGACTCGGTGCCAC
	CR34335II-Age-F	CATACCGGTCGTTTTGTCATCGCTTTTTGTCG
	CRU6/34335II-Kpn-R	CATGGTACCAAAAAAAGCACCGACTCGGTGCCAC

Table S2-II List of primers for transgenic gRNA vectors constructions used in this study

Target locus	Primer name	Primer sequence (5' – 3') Forward and Reverse
ms(3)k81	K81-KOD-F	TTACGCGGTAGTTTTAGAGCTAGAAATAGCAAGTT
	K81-KOD-R	TCAGAAATCCGAAGTATTGAGGAAAACATACCTA
yellow	yw-gRNA-KOD-F	GGTCGGCTGTGTTTTAGAGCTAGAAATAGCAAGTT
	yw-gRNA-KOD-R	ACACTCATCCGAAGTATTGAGGAAAACATACCTA
white	CR-W1-KOD-R	CTCCCTGAACCGGAGTCCTCCGAAAGTCTTCCACTCATATACGCTA
	CRW4-KOD-R	GATGGCGATACTTGGATGCCCGAAAGTCTTCCACTCATATACGCTA
	gRNA-KOD-F	GTTTTAGAGCTAGAAATAGCAAGTT
pUAST-3p3-RFP	3P3RPF-EcoRVF	CATGATATCCCGGGGATCTAATTCAATTAG
	3P3RPF-EcoRVR	CATGATATCGAGCTTCGCATGGTTTTGCC
