# Supplementary material for: Efficient Gene Knock-out and Knock-in with Transgenic Cas9 in Drosophila
Source: G3 (Bethesda). 2014 Mar 21;4(5):925–9. doi: 10.1534/g3.114.010496 (PMC4025491; doi:10.1534/g3.114.010496)
Supplement: Supporting Information [file supp_g3.114.010496_TableS3.pdf]

Table S3 List of primers for PCR check in mutations used in this study

Target locus	Primer name	Primer sequence (5' – 3') Forward and Reverse
yellow	yellow-F	CGGAGCTAATTCCGTATCCA
	yellow-R	CGCCAGGTAGCTCGTATCTC
ms(3)k81	CG14251 -F	GAGATTTCTCACTACTGCTCCTCG
	CG14251 -R	ACACGAATTGGATATGCGATAGC
white	White-Seq-F1	GGTTAGATGAGCATAACGCTTGTAG
	White-Seq-R1	CCACGCTGGATAGGAGTTGAGAT
Hisc-RA	Hisc-RA-HLF	CTAACCGGTTAGGGAGTTAGAGTGGTCGTGGC
	Hisc-RA-HLR	CAGGCGGCCGCCGTACAGCTGTAAGTCCTTGCTGA
	Hisc-RA-HRF	GGCGCGCCTTGTGGCATAGTATGAGCGATTGC
	Hisc-RA-HRR	ACTAGTTAGTTCGTATCAACACTCTACCCCAG
	Hisc-RA-F01	TTGTAAACCCAACTATCCTATCCG
	Hisc-RA-R01	CCAAGCAAATGGCAAAGGTCC
	Hisc-RA-F02	TGATGGCGTGTTGAAAGGAGAGA
	Hssc-RA-R02	GCAACTAGTGCTCTTAGCACTTTCTTG
	Hisc-RA-F03	TACGAGGAAGAATGAGACAACCA
	Hisc-RA-R03	TATAAGGACGGCACCAAAGCGC
